# The Landscape of Long Non-Coding RNA Dysregulation and Clinical Relevance in Muscle Invasive Bladder Urothelial Carcinoma

**DOI:** 10.3390/cancers11121919

**Published:** 2019-12-02

**Authors:** Haotian Shen, Lindsay M. Wong, Wei Tse Li, Megan Chu, Rachel A. High, Eric Y. Chang, Jessica Wang-Rodriguez, Weg M. Ongkeko

**Affiliations:** 1 Department of Otolaryngology-Head and Neck Surgery, University of California San Diego, La Jolla, CA 92093, USA; h9shen@ucsd.edu (H.S.); lmw010@ucsd.edu (L.M.W.); wtl008@ucsd.edu (W.T.L.); mec043@ucsd.edu (M.C.); 2 Department of Radiology, University of California San Diego, La Jolla, CA 92093, USA; rachelahigh@gmail.com (R.A.H.); ericchangmd@gmail.com (E.Y.C.); 3 Radiology Service, VA San Diego Healthcare System San Diego, La Jolla, CA 92161, USA; 4 Department of Pathology, University of California San Diego, La Jolla, CA 92093, USA; Jessica.Wang-Rodriguez@va.gov; 5 Pathology Service, VA San Diego Healthcare System, San Diego, CA 92161, USA

**Keywords:** lncRNAs, lncRNA-protein interaction, bladder carcinoma, TCGA

## Abstract

Bladder cancer is one of the most common cancers in the United States, but few advancements in treatment options have occurred in the past few decades. This study aims to identify the most clinically relevant long non-coding RNAs (lncRNAs) to serve as potential biomarkers and treatment targets for muscle invasive bladder cancer (MIBC). Using RNA-sequencing data from 406 patients in The Cancer Genome Atlas (TCGA) database, we identified differentially expressed lncRNAs in MIBC vs. normal tissues. We then associated lncRNA expression with patient survival, clinical variables, oncogenic signatures, cancer- and immune-associated pathways, and genomic alterations. We identified a panel of 20 key lncRNAs that were most implicated in MIBC prognosis after differential expression analysis and prognostic correlations. Almost all lncRNAs we identified are correlated significantly with oncogenic processes. In conclusion, we discovered previously undescribed lncRNAs strongly implicated in the MIBC disease course that may be leveraged for diagnostic and treatment purposes in the future. Functional analysis of these lncRNAs may also reveal distinct mechanisms of bladder cancer carcinogenesis.

## 1. Introduction

Bladder urothelial carcinoma is the fourth most common and eighth deadliest cancer among men, with four times as many males diagnosed than females among the approximately 80,000 annual cases in the United States [1]. One quarter to one third of bladder carcinomas invade bladder muscles and become muscle invasive bladder cancer (MIBC) [2]. This more aggressive form of bladder cancer has a greater than 10% lower survival rate than other forms and a 20–40% chance of recurrence [2]. However, the complicated molecular mechanism and diverse etiology of bladder cancer development hinder improvements in the detection and treatment of MIBC, and there has been no new treatment methods approved for over 30 years [3]. Therefore, there is a pressing need for identifying a functional biomarker for the early identification and treatment of MIBC, as well as for differentiating MIBC from non-muscle invasive bladder cancer. This study will comprehensively investigate the role of one such promising biomarker candidate, long non-coding RNAs (lncRNAs), in MIBC, using large-scale bioinformatics analyses.

Long non-coding RNAs (lncRNAs) are a category of non-coding RNAs with a length longer than 200 nucleotides [4]. In the past few years, studies of lncRNAs have revealed their roles in regulating a diverse number of critical cellular processes, including transcription and interaction with the immune system [4]. LncRNAs are implicated in both tumor suppressing and oncogenic functions in carcinogenesis, including that of bladder cancer [5]. They are attractive targets for investigation in cancer, because of their potential to serve both as diagnostic markers and as therapeutic targets. They may be excellent diagnostic markers, because there are often dramatic changes in their levels in cancer samples compared to normal samples, and they can be detected noninvasively in peripheral blood, often as part of exosomes [6]. They may also serve as useful therapeutic targets, because they may be used to regulate proteins that are not targetable, such as those that need to be upregulated or activated [6].

Extensive research has been performed on the manipulation of one or more lncRNAs in vitro, and the resulting changes in bladder carcinoma cells have been observed [7,8,9], which has led to the identification of multiple lncRNAs that are upregulated and downregulated in bladder carcinoma. However, there is still a relative lack of comprehensive analyses examining the landscape of all lncRNA expression in MIBC. Such analyses would not be possible in vitro, but could be done through computational profiling. Previous bioinformatics analyses have examined lncRNAs in MIBC using GENCODE annotations [10], but no study has performed a more comprehensive assessment of lncRNAs using LNCipedia annotations. Finally, most of the previous lncRNA studies examined known pathways involved in tumor initiation and proliferation and are limited by the number of samples or patients they investigated [8,9]. In this study, we utilized RNA-sequencing data of MIBC samples from 406 patients, downloaded from The Cancer Genome Atlas (TCGA) database. We discovered a panel of 20 lncRNAs significantly dysregulated in MIBC that are also significantly associated with patient survival and clinical variables. We correlated lncRNA expression with genomic alterations to investigate possible mechanisms for lncRNA dysregulations, and we analyzed the lncRNAs’ association with cancer and immune-associated pathways and cancer-related signatures. Collectively, our data suggest that these 20 lncRNAs may serve as a potentially useful biomarker panel for predicting patient prognosis, clinical characteristics, and genomic profiles based on their expressions. As these lncRNAs are clinically relevant, they should also be investigated as potential treatment targets.

## 2. Results

### 2.1. Identifying Dysregulated lncRNAs in Bladder Carcinoma

**Figure 1:**
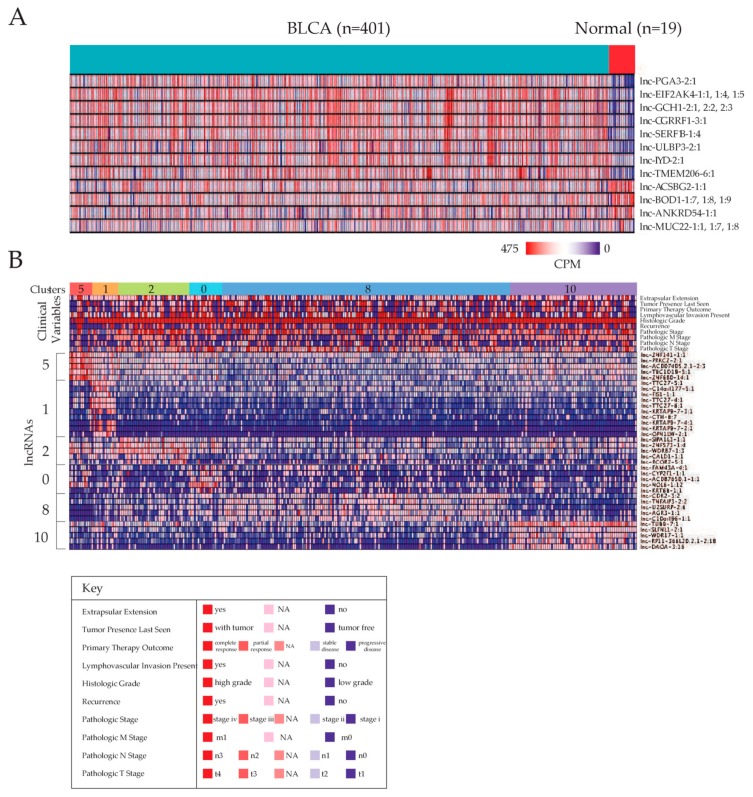

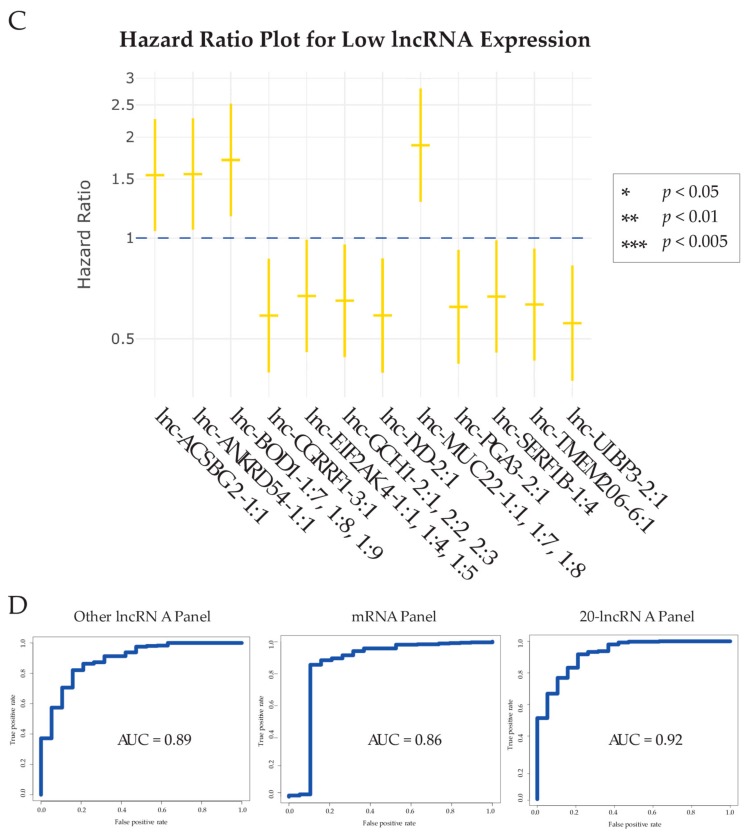
Summary of differential expression and patient survival results. (**A**) Heat map of significant differentially expressed long non-coding RNAs (lncRNAs) (FDR < 1 × 10^−5^, |log(fold change)| > 1) when comparing bladder urothelial carcinoma (BLCA) samples to adjacent normal samples. (**B**) Clustering of patients based on lncRNA expression landscape. The top portion of the heat map represents the patient clinical parameter. The bottom heat map depicts lncRNAs most prominently expressed in each cluster. (**C**) Hazard ratio plots of significant differentially expressed lncRNAs for patients in a low expression group (below median expression) for each lncRNA. All lncRNAs presented also significantly correlate with patient survival and one or more clinical variables. (**D**) Receiver operating characteristic (ROC) curves illustrating the power of three different expression panels in discriminating muscle invasive bladder cancer (MIBC) samples from normal samples.

To evaluate the landscape of lncRNA dysregulation in MIBC patients, lncRNA expression data of cancer tissues from 406 patients were downloaded from the TCGA database and compared to lncRNA read counts of 19 normal tissues. Differential expression analysis was performed with a negative binomial model, and 3191 lncRNAs were identified to be dysregulated in cancer tissue compared to normal tissue (Supplementary Table S1). An lncRNA was considered to be dysregulated if the false discovery rate (FDR) of edgeR exact test was less than 1 × 10^−5^, and its expression fold change in cancer tissues was greater than 2 or less than −2, compared to normal tissues. The low FDR cutoff was chosen to achieve high confidence of differential expression and focus on the most significantly dysregulated lncRNAs. The very large number of lncRNAs dysregulated, even at a strict significance cutoff, corroborates with previous reports that the expression of lncRNAs changes dramatically in cancer tissue compared to normal tissue [6]. All differentially expressed lncRNAs were analyzed for association with patient survival and clinical variables to identify the transcripts most implicated in MIBC prognosis, which would be ideal candidates for diagnostic or treatment purposes. After these analyses, we identified a core set of 20 lncRNAs with significant differential expression, survival correlation, and clinical variable correlation that will be the focus of subsequent sections. The 20 lncRNAs are grouped into 12 sets with distinct expression patterns (lncRNAs adjacent to each other, such as *lnc-EIF2AK4-1:1*, *lnc-EIF2AK4-1:4*, and *lnc-EIF2AK4-1:5*, have the same expression pattern in nearly all patients), and their differential expression is visualized in Figure 1A. Although the expression of lncRNAs is low compared to that of protein-coding genes, their expressions ranges are still detectable and comparable to the expression ranges of some protein-coding genes implicated in bladder cancer, namely *GSTM1*, *TSC1*, *HOXB2*, *NAT2*, and *APC* (Supplementary Figure S1).

### 2.2. Stratification of Patients Based on lncRNA Expression Landscapes

We explored the entire landscape of lncRNA expression in the 406 patients and clustered the patients into groups based on patterns of lncRNA expression, using a combination of lncRNAs instead of single lncRNAs. This clustering would aggregate the predictive power of single-lncRNA expression into lncRNA panel predictors, and at the same time allow us to discover patient groups that lncRNA expression would be more likely to discriminate. We discovered 11 clusters of patients that are significantly enriched for patients with particular clinical variables, suggesting that lncRNA expression can effectively stratify patients based on clinical variables (Figure 1B). Clusters 2 and 0 are enriched for patients with tumor lymphovascular invasion, while clusters 1 and 10 are enriched for patients without lymphovascular invasion. Clusters 5 and 1 are enriched for pathologic stage II patients, while cluster 2 is enriched for pathologic stage IV patients. Clusters 5, 1, and 8 are enriched for pathologic T stage 2 patients, while clusters 2 and 10 are enriched for pathologic T stage 3 patients. In summary, cluster 2 seem to be composed of patients with more aggressive forms of MIBC, while clusters 5 and 1 seem to be composed of patients with the best prognosis. We further identified the specific lncRNAs or sets of lncRNAs that are most able to discriminate different patient clusters from each other (Figure 1B). Clusters for which we could not derive a set of marker lncRNAs are not displayed in Figure 1B.

### 2.3. Analyzing Association between lncRNA Dysregulation and Bladder Carcinoma Patient Survival

We correlated patient survival rate with the expressions of the 3191 differentially expressed lncRNAs using the Cox proportional hazards regression model. The expression levels of only 257 lncRNAs were found to have significant correlation with patient survival rates (*p* < 0.05, Supplementary Table S2), and only 20 of these lncRNAs are correlated with survival in the same direction, as is expected from dysregulation (i.e., if the lncRNA is upregulated in MIBC, high expression of the lncRNA should correlate with poor survival). The low number of lncRNAs that exhibit survival correlation suggests that most dysregulated lncRNAs may have no significant function in influencing MIBC progression. However, certain lncRNAs were found to correlate strongly with the clinical phenotype, and are most likely functionally significant.

We visualized the correlation of lncRNA expressions with survival using Kaplan–Meier plots, which were produced using right-censored observations of patient survival data to predict the survival function. Kaplan–Meier plots were generated for the 20 core lncRNAs, which all exhibited significant survival correlations consistent with their direction of expression dysregulation, as well as their direction of correlation with clinical variables, described in the next section (Supplementary Figure S2). Eight downregulated lncRNAs—*lnc-ACSBG2-1:1*; *lnc-ANKRD54-1:1*; *lnc-BOD1-1:7*, -*1:8*, and -*1:9*; and lnc-*MUC22-1:1*, -*1:7*, and -*1:8*—demonstrated correlation between lower patient survival rate with lower expression of lncRNA, indicating a potential tumor-suppressing role for these lncRNAs. Twelve upregulated lncRNAs—*lnc-CGRRF1-3:1*; *lnc-EIF2AK4-1:1*, -*1:4*, and -*1:5*; *lnc-GCH1-2:1*, -*2:2*, and -*2:3*; *lnc-IYD-2:1*; *lnc-PGA3-2:1*; *lnc-SERF1B-1:4*; *lnc-TMEM206-6:1*; and *lnc-ULBP3-2:1*—displayed correlation between lower patient survival rate and higher expression of lncRNA, suggesting potential oncogenic properties. Calculation of hazard ratios for patients in the low expression group of each lncRNA indicates that the patient is generally between 1.5 to 2.0 times more likely to die if the lncRNA is downregulated, and between 0.75 and 0.50 times more likely to die if the lncRNA is upregulated (Figure 1C).

### 2.4. Comparison of the Diagnostic Power of our lncRNA Panel with Other Expression Panels

We evaluated the ability of our lncRNA panel to discriminate bladder cancer samples from adjacent samples using leave-one-out cross validation, which derives a predictive function using all datapoints but one, and then uses the function to predict whether the left-out datapoint was a cancer or normal sample. Visualization through the receiver operating characteristic (ROC) curves indicated that the area under the curve (AUC) was the greatest for our lncRNA panel out of three expression panels, suggesting that our panel has the greatest diagnostic power (Figure 1D). We obtained the external lncRNA panel from Seitz et al. [11], and derived the mRNA expression panel by locating 40 genes most implicated in bladder cancer through a literature search.

### 2.5. Correlating Dysregulated lncRNA Expression Levels with Clinical Variables

**Figure 2:**
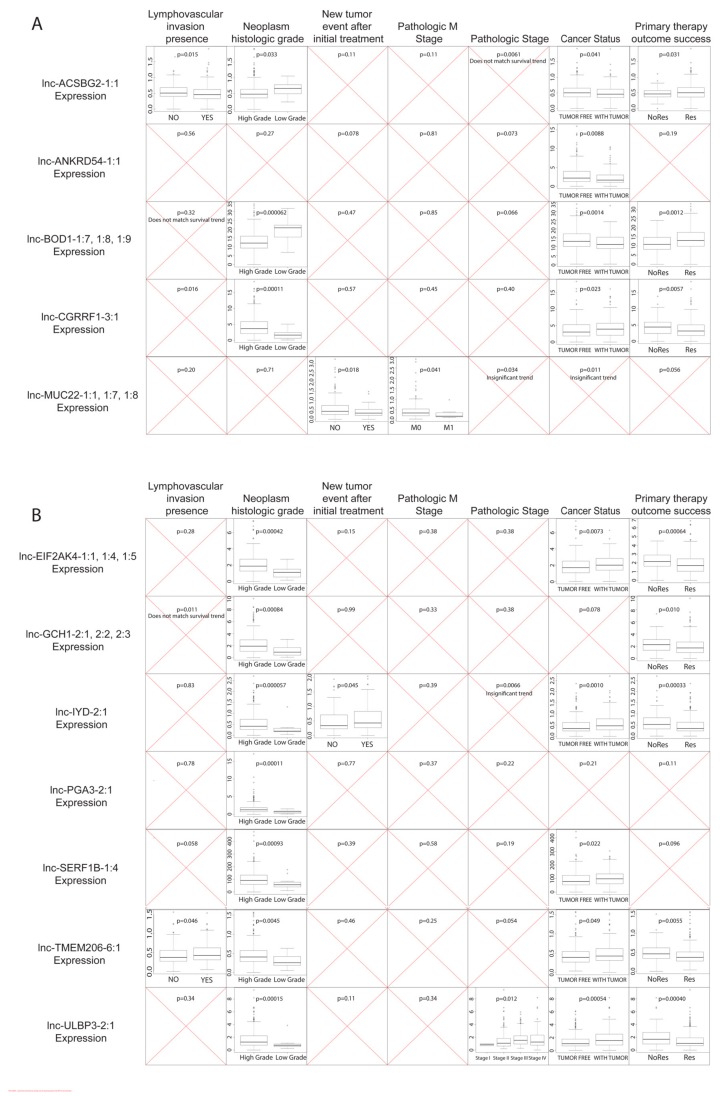
Clinical variable correlation with expression of key lncRNAs. Boxplots depict significant correlations (Kruskal–Wallis tests; *p* < 0.05) with clinical variables for (**A**) significantly upregulated lncRNAs and (**B**) significantly downregulated lncRNAs that correlated with patient survival. The red crosses indicate non-significant correlations. An explanation for non-significance is given below the stated *p*-value if it is <0.05.

We correlated the expression of dysregulated lncRNAs with multiple clinical variables, including pathologic TNM stages (tumor, node, and metastasis), pathologic stage, presence of tumor at last follow-up (cancer status), histologic grade, therapy success, new tumor event after therapy, and lymphovascular invasion, using the Kruskal–Wallis test (*p* < 0.05; Figure 2). We discovered that most of the core 20 lncRNAs exhibited correlations with histologic grade, primary therapy outcome success, and cancer status, indicating that survival-associated, dysregulated lncRNAs are associated with cancer cell phenotype and ease of elimination. On the other hand, clinical variables like invasion, pathologic stages, and new tumor events are not highly associated with lncRNA expression. For half of the downregulated lncRNAs, namely *lnc-ACSBG2-1:1* and *lnc-BOD1-1:7*, -*1:8*, and -*1:9*, high lncRNA expression levels correlated with primary therapy outcome success and lower tumor grade, which further suggest potential tumor-suppressing roles. High expression of all the above lncRNAs plus *lnc-ANKRD54-1:1*, which was also downregulated in MIBC, are associated with a lack of tumor presence at last follow-up. For upregulated lncRNAs, higher expression of *lnc-CGRRF1-3:1*; *lnc-EIF2AK4-1:1*, -*1:4*, and -*1:5*; *lnc-GCH1-2:1*, -*2:2*, and -*2:3*; *lnc-IYD-2:1*; *lnc-TMEM206-6:1*; and *lnc-ULBP3-2:1* were associated with both lack of primary therapy outcome success and higher tumor grade, indicating the potential oncogenic roles of these lncRNAs in MIBC.

### 2.6. Correlating Expressions of Key lncRNAs to Cancer-Associated Gene Sets

Gene set enrichment analysis (GSEA) was used to correlate the expression of the 20 key lncRNAs to that of genes in biologic pathways and oncogenic signatures. Canonical pathways were drawn from curated databases, including Biocarta, Reactome, and the Kyoto Encyclopedia of Genes and Genotype (KEGG), and included genes that participate in specific biological processes. Only pathways relevant to tumor development and progression, including cancer process-associated and immune-associated pathways, were examined. Oncogenic signatures are gene sets containing genes with the most significant expression changes after in vitro experiments manipulating the expression of a cancer-associated gene.

**Figure 3:**
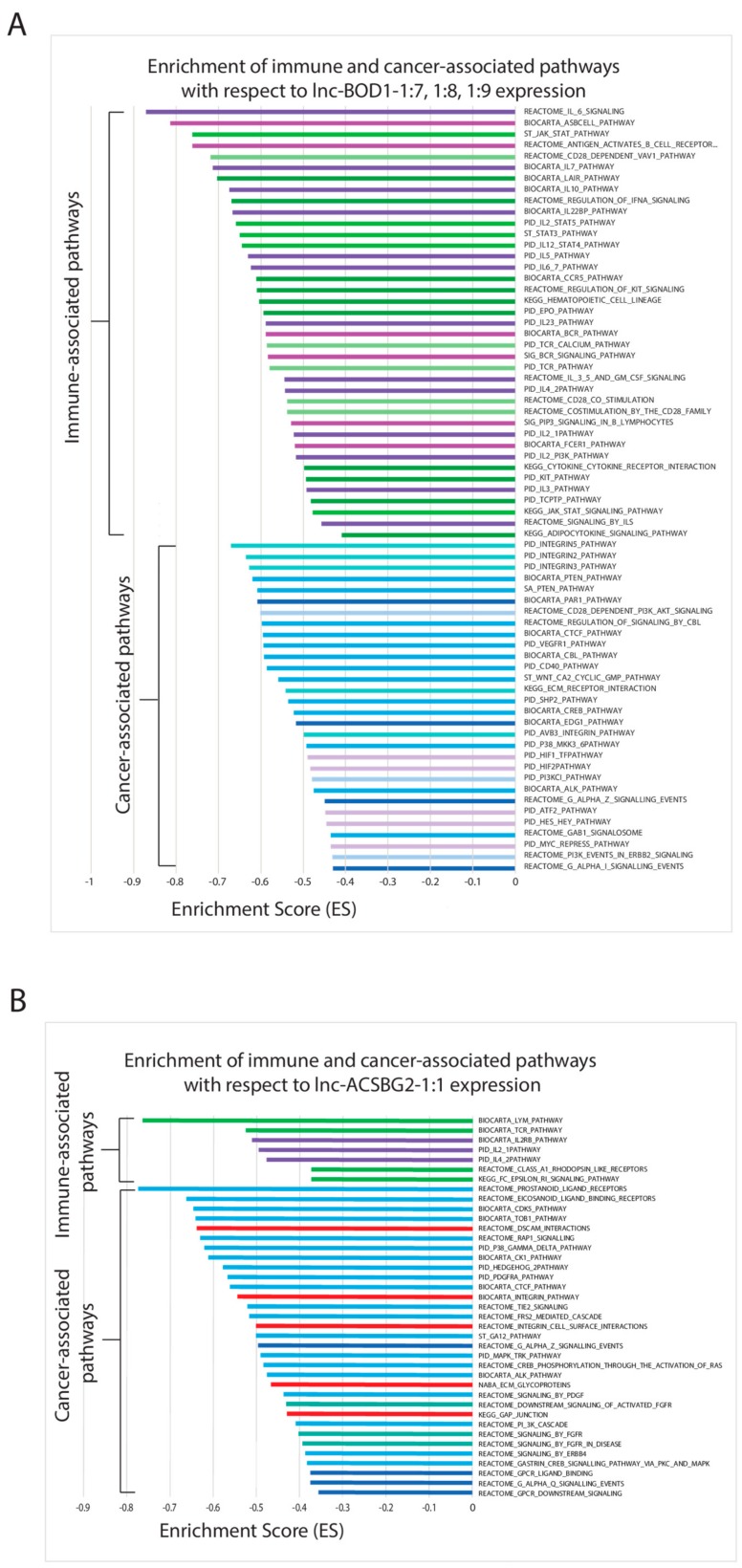

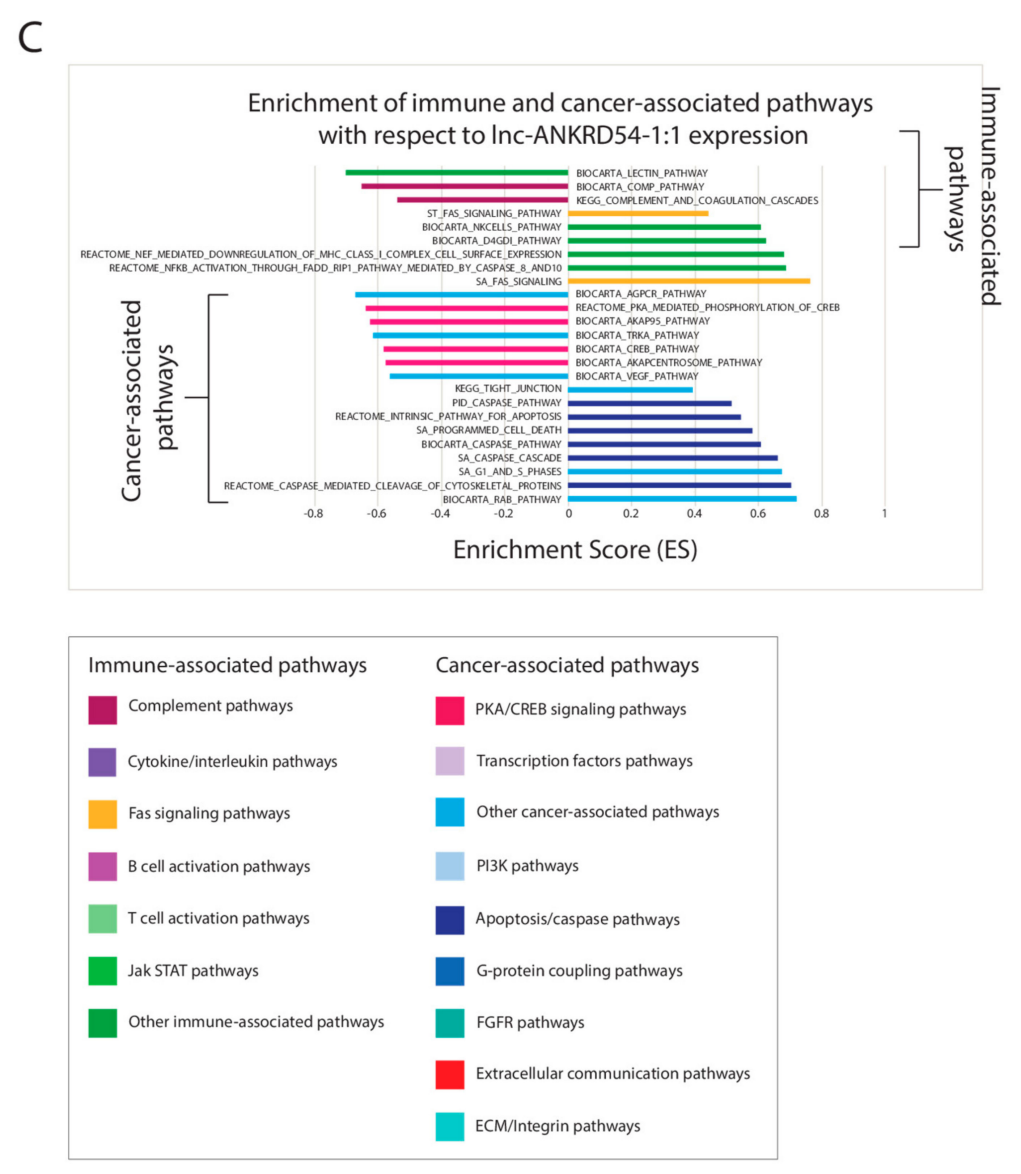
Canonical pathways filtered for cancer-associated pathways and immune-associated pathways (C2) from gene set enrichment analysis (GSEA) (nominal *p* < 0.05) for the significantly downregulated lncRNAs including (**A**) lnc-BOD1-1:7, 1:8, 1:9; (**B**) lnc-ACSBG2-1:1; and (**C**) lnc-ANKRD54-1:1. Significantly downregulated lncRNAs with fewer than 15 cancer- and immune-associated pathways are placed in the Supplementary Materials (Supplementary Figure S3A).

The different lncRNAs correlate with the activities of immune- and cancer-associated pathways in different ways. For downregulated lncRNAs, *lnc-ACSBG2-1:1* and *lnc-BOD1-1:7*, -*1:8*, and -*1:9* exhibit similar landscapes of pathway enrichment, where their low expression correlates with a high expression of genes in immune- and cancer-associated pathways, as represented by a negative enrichment score (Figure 3). The correlation of low lncRNA expression with greater cancer-associated activity lends further evidence that the lncRNAs function as tumor suppressors. Multiple pathways related to fibroblast growth factor receptor (FGFR) signaling, G-protein-coupled receptors, and extracellular communication are implicated with *lnc-ACSBG2-1:1*, while pathways related to the extracellular matrix, G-protein-coupled receptors, and oncogenic transcription factors are implicated with *lnc-BOD1-1:7*, -*1:8*, and -*1:9* (Figure 3). Interestingly, immune-associated pathways are also upregulated with low lncRNA expression, suggesting that some lncRNAs that are functionally important in bladder cancer may be immunogenic. Cytokine, interleukin, and B-cell signaling comprise a large number of implicated immune-associated pathways. For *lnc-ANKRD54-1:1*, which is also downregulated in MIBC, cancer- or immune-associated pathways are not enriched in the same direction. Instead, low *lnc-ANKRD54-1:1* expression is associated with the upregulation of important cancer pathways (mostly involved in protein kinase A and cAMP response element-binding protein signaling) and the downregulation of many immune-associated and apoptosis-related pathways. Finally, the downregulated lncRNAs *lnc-MUC22-1:1*, -*1:7*, and -*1:8* exhibit correlation of low expression with low immune-associated activities and low cell–cell junction activities (Supplementary Figure S3A).

**Figure 4:**
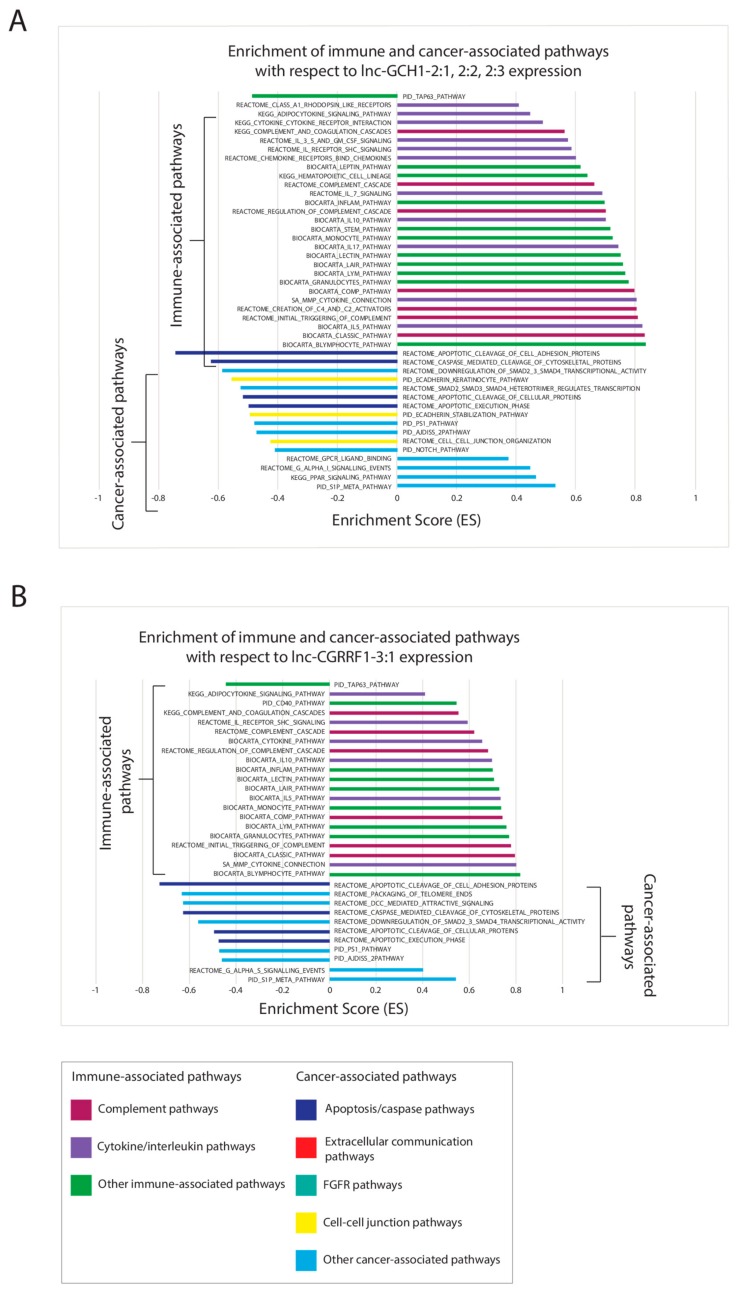
Canonical pathways filtered for cancer-associated and immune-associated pathways (C2) from gene set enrichment analysis (nominal p < 0.05) for significantly upregulated lncRNAs including (**A**) lnc-GCH1-2:1, 2:2, 2:3; and (**B**) lnc-CGRRF1-3:1. Significantly upregulated lncRNAs with fewer than 15 cancer- and immune-associated pathways are placed in the Supplementary Materials (Supplementary Figure S3B).

On the other hand, for upregulated lncRNAs, *lnc-CGRRF1-3:1* and *lnc-GCH1-2:1*, -*2:2*, and -*2:3* share nearly identical correlations with pathways, and we found that high lncRNA expression for these four lncRNAs leads to increased immune activity, but decreased apoptosis (Figure 4). The complement pathways and cytokine/interleukin signaling pathways account for the majority of immune activities that are upregulated. Other upregulated lncRNAs exhibit very little significant correlation between lncRNA expression and pathways in comparison (Supplementary Figure S3B). 

Immune-associated pathways that were associated with the most lncRNAs include the complement pathway (BIOCARTA_COMP_PATHWAY), lectin pathway (BIOCARTA_LECTIN_PATHWAY), and local acute inflammatory response pathway (BIOCARTA_LAIR_PATHWAY), while the cancer-associated pathways that were associated with the most lncRNAs include the caspase pathway (REACTOME_CASPASE_MEDIATED_CLEAVAGE_OF_CYTOSKELETAL_PROTEINS), Wnt signaling pathway (ST_WNT_CA2_CYCLIC_GMP_PATHWAY), cell adhesion degradation (REACTOME_APOPTOTIC_CLEAVAGE_OF_CELL_ADHESION_PROTEINS), and the SMAD pathway (REACTOME_DOWNREGULATION_OF_SMAD2_3_SMAD4_TRANSCRIPTIONAL_ACTIVITY).

**Figure 5:**
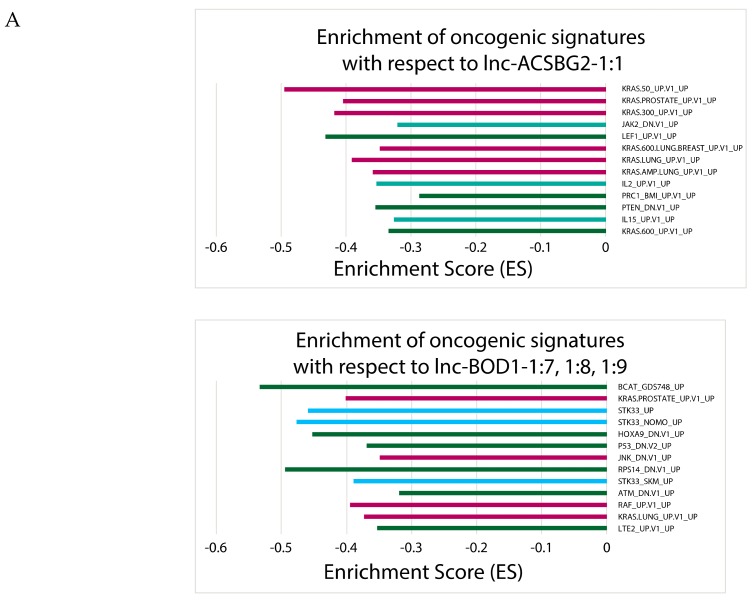

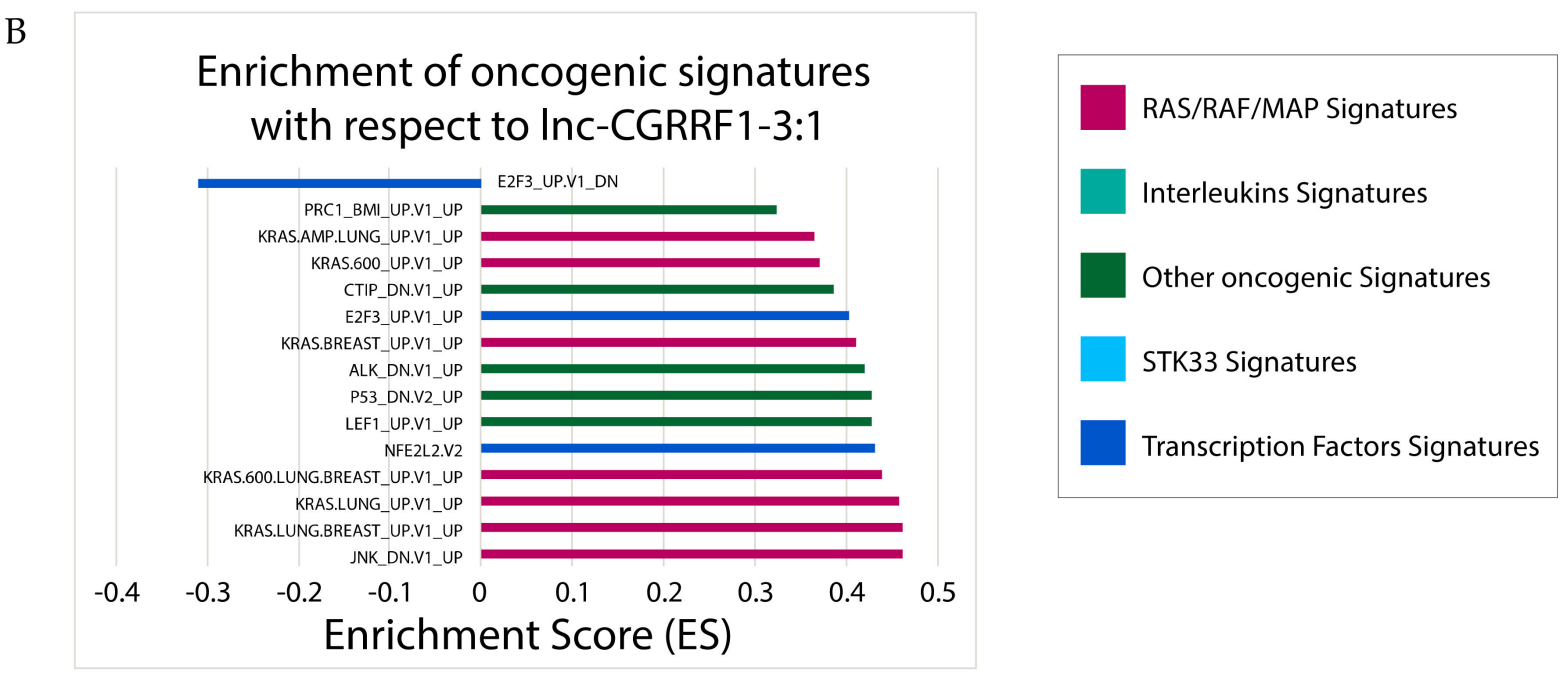
Significant oncogenic signatures (C6) from GSEA (nominal *p* < 0.05). Barplots showing (**A**) significantly downregulated lncRNAs and (**B**) significantly upregulated lncRNAs that correlate with patient survival and clinical variables. All bars extending towards the left indicate negative enrichment of pathway activity to lncRNA expression, such that higher pathway activity corresponds to lower lncRNA expression. Significantly upregulated or downregulated lncRNAs with fewer than 10 oncogenic signatures are placed in Supplementary Materials (Supplementary Figure S4).

After correlating lncRNA expressions to oncogenic signatures, the lncRNAs that correlated to the most oncogenic signatures included *lnc-CGRRF-3:1*, *lnc-ACSBG2-1:1*, and *lnc-BOD1-1:7*, -*1:8*, and -*1:9*, whereas the lncRNAs that did not correlate with any oncogenic signatures included *lnc-EIF2AK4-1:1*, -*1:4*, and -*1:5*, as well as *lnc-GCH1-2:1*, -*2:2*, and -*2:3*; *lnc-IYD-2:1*; *lnc-MUC22-1:1*, -*1:7*, and -*1:8*; and *lnc-PGA3-2:1* (Figure 5, Supplementary Figure S4). Oncogenic signatures that were associated with the most lncRNAs are KRAS-related (KRAS.LUNG_UP.V1_UP and KRAS.AMP.LUNG_UP.V1_UP). Five out of the eight downregulated lncRNAs were associated with oncogenic processes, while only 4 out of 12 upregulated lncRNAs were associated with oncogenic processes. Collectively, our GSEA correlations suggest that downregulated lncRNAs are more likely to induce changes on the multi-gene or pathway level than upregulated lncRNAs are.

### 2.7. Investigating Genomic Alterations’ Association with lncRNA Expression

The repeated evaluation of variables’ conditional entropy and redundancy (REVEALER) algorithm, developed by Kim et al. [12], was used to computationally correlate all genomic alterations (or mutations and copy number alterations (CNAs)) present in MIBC samples to lncRNA expression. This exhaustive process aims to identify genomic alterations that are functionally responsible for the range of lncRNA expression values in the patient samples, as opposed to associations with the general potential driving processes of bladder cancer that we performed using GSEA. Utilizing the concepts of mutual information and information coefficient from information theory, REVEALER calculates a conditional information coefficient (CIC) for each genomic alteration event. When the absolute value of the CIC is above around 0.30, the correlation between the event and lncRNA expression is reasonably established, based on test data sets [13].

**Figure 6:**
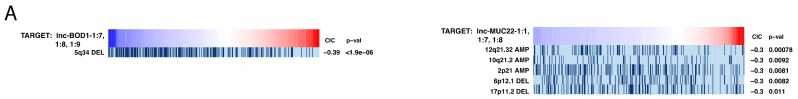

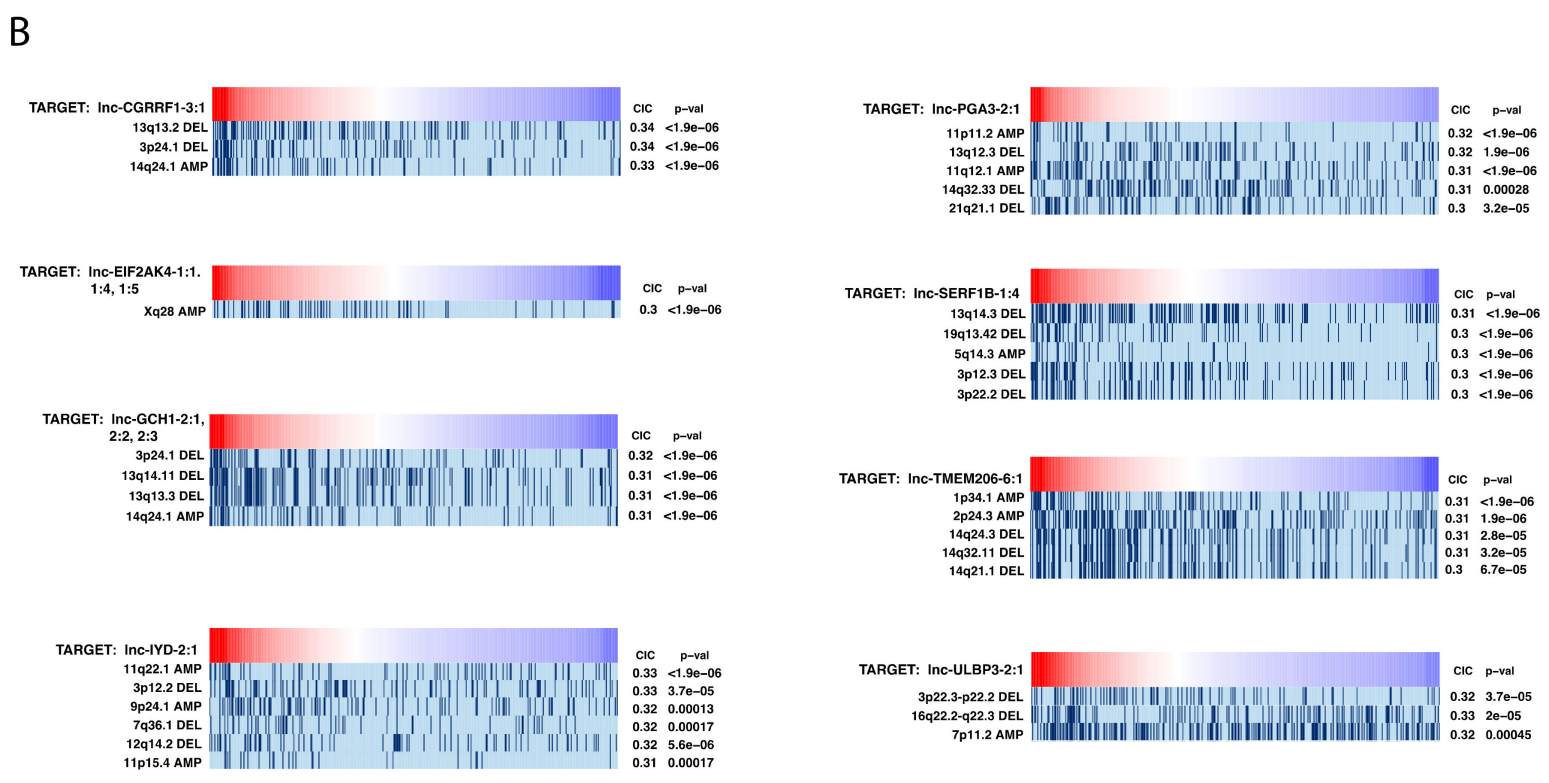
Association of genomic alterations with the expression of (**A**) significantly downregulated lncRNAs and (**B**) significantly upregulated lncRNAs that correlated with patient survival and clinical variables using repeated evaluation of variables’ conditional entropy and redundancy (REVEALER) (|CIC| > 0.3). The gradient bar visualizes the expression distribution of the lncRNA. The dark red extreme represents the highest expression, whereas the dark blue extreme represents the lowest expression.

We discovered that all upregulated key lncRNAs are associated with one or more genomic alteration events, while downregulated lncRNAs *lnc-ACSBG2-1:1* and *lnc-ANKRD54-1:1* do not correlate with any genomic alterations (Figure 6). No mutations correlated with lncRNA expression, so copy number changes are primary genomic alterations that alter lncRNA expression. Several CNA regions correlate with the expression of multiple lncRNAs. Deletion of 13q12-14, deletion or amplification of 14q24-32, and deletion of the 3p22-24 locus all associate with the expression of four sets of upregulated lncRNAs (Figure 6B).

We also identified all genes dysregulated in MIBC located within CNA regions correlated with lncRNA expressions, and noted whether these genes may be oncogenes, tumor suppressors, or immune-associated (IA) genes, according the COSMIC Cancer Gene Census [14] (Supplementary Table S3). Only a small fraction of genes in these regions are any of the above, and we also found only four dysregulated genes within these CNA regions that were correlated with survival (Supplementary Table S4, Cox regression, *p* < 0.05). Survival correlations were obtained from OncoLnc [15]. These results suggest that the copy number changes may be directly related to lncRNA dysregulation, rather than the lncRNA dysregulations being a passenger effect.

### 2.8. Investigating the Potential Role of lncRNAs as Inhibitors of MicroRNAs (MiRNAs)

LncRNAs have been known to serve as decoys or “sponges” for microRNAs (miRNAs) to bind instead of their target messenger RNAs (mRNAs), effectively suppressing the function of the miRNAs [16]. We analyzed the potential for miRNAs significantly dysregulated in MIBC to bind to the key lncRNAs using the LncBase tool in DIANA tools, which outputs an interaction score between a given lncRNA and miRNA pair [12]. We discovered that *lnc-BOD1-1:7*, -*1:8*, and -*1:9*; *lnc-EIF2AK4-1:4*, *1:5*; and *lnc-MUC22-1:1* are predicted to interact with several miRNAs dysregulated in MIBC, with most of these miRNAs being upregulated in MIBC (Supplementary Table S5). Therefore, these lncRNAs can potentially neutralize the effects of these miRNAs’ upregulation.

## 3. Discussion

Long non-coding RNAs are known to play important roles in cancer pathogenesis pathways, including promoting cancer initiation and maintaining cancer development, illustrating their great potential as diagnostic biomarkers or therapeutic targets for cancer [17]. Many lncRNAs have been implicated in the regulation of proteins through direct interaction [3]. In this study, we comprehensively examined lncRNA dysregulation in MIBC in the context of clinical relevance, potential interaction with proteins, and correlation with genomic alterations in MIBC, in order to identify the most significant lncRNAs implicated in bladder cancer.

We identified 3191 lncRNAs, among 107,039 known lncRNAs, to be differentially expressed in a patient cohort of 406 MIBC samples vs. adjacent normal samples (FDR < 0.05). Out of these lncRNA candidates, we found 20 key lncRNAs to be the most critically involved in MIBC, based on correlation with patient survival rates (univariate Cox regression analysis, *p* < 0.05) and association with selected clinical variables (Kruskal–Wallis test, *p* < 0.05). To the best of our knowledge, none of these 20 lncRNAs have been found to be implicated in bladder cancer. The lack of overlap between the lncRNAs we discovered and the lncRNAs reported in the previous manuscript is partly due to many studies documenting only one or a few lncRNAs implicated in bladder cancer [10,18]. Because we are examining a larger number of lncRNAs than most papers, the lncRNAs discovered by other studies could be insignificant in our study after multiple comparison correction. However, we did discover that several lncRNAs found to be significant in Wen et al. [18] are also significantly dysregulated in our analysis, although they were not part of the 20 key lncRNAs in our study. We have investigated a significantly larger set of lncRNAs than similar studies profiling lncRNAs in bladder cancer by using lncRNA annotations from LNCipedia, which seeks to capture all known lncRNA transcripts by compiling and standardizing transcripts discovered from different literature sources [19]. Studies using GENCODE annotations would only examine 19,812 transcripts, compared to the 107,039 transcripts in LNCipedia [10]. Previous studies have identified that lncRNAs like *LINC00460*, *UCA1*, *LINC00958*, *LINC01296*, *SNH12*, and *DUXAP8* are implicated in bladder cancer, but did not examine the entire landscape of lncRNA transcripts [11,18,20,21].

We then used GSEA to determine which canonical pathways and oncogenic signatures were most associated with the 20 clinically significant lncRNAs. When performing GSEA for canonical pathways using the C2 gene set, we found that some lncRNAs, including *lnc-BOD1-1:7*, -*1:8*, and -*1:9*, as well as *lnc-GCH1-2:1*, -*2:2*, and -*2:3* were associated with many immune- and cancer-associated pathways. Other lncRNAs were mainly correlated with either immune-associated pathways, with *lnc-CGRRF1-3:1* as an example, or cancer-associated pathways, with *lnc-ACSBG2-1:1* and *lnc-ANKRD54-1:1* as examples. Finally, lncRNAs such as *lnc-EIF2AK4-1:1*, -*1:4*, and -*1:5*, as well as *lnc-SERF1B-1:4* were not correlated with many of the immune-associated or cancer-associated pathways. For determining which oncogenic signatures were most associated with the 20 key lncRNAs, we performed GSEA using the C6 gene set, and found that *lnc-CGRRF-3:1*, *lnc-ACSBG2-1:1*, and *lnc-BOD1-1:7*, -*1:8*, and -*1:9* correlated with the most oncogenic signatures. Notably, *lnc-BOD1-1:7*, -*1:8*, and -*1:9* were highly correlated with both pathways’ gene sets and oncogenic signatures. In terms of pathways, those that are associated with the greatest number of lncRNAs include pathways involved in apoptosis and the downregulation of genes *SMAD2/3* and *SMAD4*, complement pathways, and the Wnt/Ca^2+^ pathway. With regards to signatures, KRAS oncogenic signatures were found to have the most significant association with lncRNAs.

Finally, to understand possible causes of lncRNA dysregulation, we used REVEALER to correlate lncRNA expression with genomic alterations in MIBC. Interestingly, we found that none of the 20 significant lncRNAs were correlated with any mutations, and instead were only correlated with genomic amplifications and deletions. More upregulated lncRNAs are associated with genomic alterations than are downregulated lncRNAs, and several CNA hotspots, including 13q12-14, 14q24-32, and 3p22-24, are correlated with the expression of multiple upregulated lncRNAs. All the above loci have been previously implicated in bladder cancer risk [22,23,24].

## 4. Materials and Methods

### 4.1. lncRNA-Sequencing Datasets and Clinical Data

RNA-sequencing datasets and clinical data for 406 bladder urothelial carcinoma and 19 adjacent solid normal tissue samples were obtained from The Cancer Genome Atlas (TCGA) (https://tcga-data.nci.nih.gov/tcga, accessed 17 April 2017). All cases were muscle-invasive urothelial carcinoma, and the TCGA identifications (IDs) for all cases used are listed in Supplementary Table S6. The majority of samples were obtained using transurethral resection. The remaining were obtained through endoscopic biopsy, cystectomy, or cystoprostatectomy. Clinical data for each patient sample, including survival data, stage data, and pathologic data, were obtained from the Broad Institute Firehose (https://gdac.broadinstitute.org, accessed 17 April 2017).

The lncRNA read counts were generated from RNA-sequencing datasets via BEDtools coverageBed (https://github.com/arq5x/bedtools2, accessed 17 May 2017), using lncRNA annotation files obtained from LNCipedia version 3.1 (http://lncipedia.org/, accessed 17 May 2017), a database curating over 100,000 lncRNA transcripts from sources including the Broad Institute, Ensembl, Gencode, Refseq, and NONCODE.

### 4.2. lncRNA Differential Expression Analyses

lncRNA read counts were imported into edgeR (v3.5, Bioconductor), and lowly-expressed lncRNAs (counts-per-million < 1 in more than the number of samples in the smaller cohort) were filtered from the analysis [25]. Following TMM (trimmed mean of M-values) normalization, the exact test was applied to identify significantly differentially expressed lncRNAs in MIBC tumor tissues versus normal tissues (FDR < 0.05). All lncRNAs identified as differentially expressed in each edgeR comparison were retained as candidates for further analysis.

### 4.3. Clustering of Patients Using lncRNA Expression Profiles

The full set of lncRNA expression profile for each cancer sample, including around 13,000 lncRNAs after lowly expressed transcripts were filtered, was used for patient clustering. Clustering was done with *k*-means, and the elbow method was used to determine the optimal number of clusters, which was 11. Sets of lncRNAs that could serve as markers for each cluster were found through differential expression of samples within the cluster vs. all samples outside the cluster. Clusters for which this set of marker lncRNAs could not be found are not displayed in Figure 1.

### 4.4. Receiver Operating Characteristic Curve Generation for the Diagnostic Abilities of Expression Panels

ROC curves were generated by plotting the result of a prediction through the leave-one-out cross validation algorithm, in which a predictive function was created using all but one sample, and the function was then used to predict whether the left-out sample was cancerous or normal. This process is repeated until all samples have been tested as the one left-out. The R package brglm (https://cran.r-project.org/web/packages/brglm/, accessed 25 October 2019) was used to create a generalized linear model as the predictive function in cross-validation.

### 4.5. Association of Candidate lncRNAs with Patient Survival and Clinical Variables

Survival analyses were performed using the Kaplan–Meier model, with lncRNA expression in MIBC tumors designated as a binary variable based on expression above or below the median. The time of the sensor was designated as days to last follow-up or days to death. Univariate Cox regression analysis was used to identify candidates significantly associated with patient survival (*p* < 0.05). Survival-correlated lncRNAs between cancer and normal cohorts were evaluated for clinical significance. Employing the Kruskal–Wallis test, we investigated the lncRNA association with a neoplasm histologic grade, pathologic stages, and lymphovascular invasion, using clinical data and lncRNA expression values (counts-per-million) from MIBC patients. The Union for International Cancer Control/American Joint Committee on Cancer (UICC/AJCC) TNM staging system was used for pathological stages. In TNM staging analysis, patients were grouped together based on the priority of cancer development (i.e., M > N > T; patients were always grouped with their highest possible development). Patients with no available information for a given characteristic were filtered from analyses involving that variable.

### 4.6. Identification of Gene Sets Associated with Clinically Significant lncRNA Expression

Canonical biologic pathways (C2:CP gene sets) and oncogenic signatures (C6 gene sets) were downloaded from the Molecular Signatures Database (http://software.broadinstitute.org/gsea/msigdb/index.jsp, accessed 06 October 2017 ) [26]. All gene sets were analyzed for enrichment with respect to the gene expressions of all clinically significant lncRNAs, using gene set enrichment analysis (GSEA) [27]. The lncRNA expressions were inputted as a continuous variable for the phenotype input of GSEA, while expression of all genes in counts per million (CPM) were used as the gene expression dataset input. Pearson correlation was used to correlate lncRNA expression with gene expression to produce a ranked list of lncRNA-gene co-expression. Significant GSEA associations between an lncRNA and a gene set (*p* < 0.05) were then filtered so that only canonical pathways with cancer and immune relevance were retained. Only oncogenic signatures enriched in a direction consistent with the direction of the signature were retained. For example, if the signature contained genes upregulated after manipulation of a cancer-related gene, only plots where upregulation of the signature genes correlates with the dysregulation of an lncRNA were retained.

### 4.7. Information Coefficient-Based Correlation of lncRNA Expression with Genomic Alterations

The annotation files for mutation and CNAs were compiled into a binary input file for the program REVEALER (Repeated Evaluation of VariablEs conditionAL Entropy and Redundancy), designed to computationally identify a set of specific CNAs and mutations most likely responsible for the change in activity of a target profile [13]. The target profile was defined in our study to be lncRNA expression. In order to identify a set of the most relevant genomic alterations, REVEALER runs multiple iterations of the correlation algorithm, with the genomic feature exhibiting the strongest correlation in each run serving as a seed for the successive run. We set the maximum number of iterations to three. A seed is a particular mutation or copy number gain or loss event that most likely accounts for the target activity. When given a seed, REVEALER will focus correlation only on patients with altered target activity not accounted for by the seed. Since we do not know which genomic alteration is responsible for the dysregulation of each lncRNA, we set the seed for the first iteration to null. We set the threshold of genomic features to input to features present in less than 75% of all samples.

### 4.8. LncBase Prediction of MiRNA–LncRNA Interaction

The web-based DIANA-LncBase software (v.2) [12] was used to predict miRNA–lncRNA binding. The threshold for significant interaction was set at 0.7 for the miRNA-target gene (miTG) score. Only miRNAs found to be differentially expressed in MIBC, as we described in our previous publication [28], were included in the analysis.

## 5. Conclusions

Through large-scale bioinformatics analysis with a large sample size, we seek to prioritize the significance of lncRNAs implicated in MIBC pathogenesis and progression. In this study, we identified a panel of 20 significantly dysregulated lncRNAs that may be the most clinically important for MIBC in a 400-patient database. The expression of the 20 lncRNAs were discovered to correlate with patient survival, clinical phenotype, genomic alterations, immune- and cancer-associated pathways, and oncogenic signatures. By demonstrating strong correlations between an lncRNA panel and clinically relevant variables or molecular phenotypes, we provide an argument for the attractive potential of using lncRNAs as both biomarkers and treatment targets for MIBC.

## Supplementary Materials

The following are available online at https://www.mdpi.com/2072-6694/11/12/1919/s1, Figure S1: Comparisons of MIBC gene expression to lncRNA expression, Figure S2: Correlations of patient survival with lncRNA expression, Figure S3: Other lncRNA-canonical pathways correlations, Figure S4: Other lncRNA-oncogenic signatures correlations, Table S1: Differentially expressed lncRNAs, Table S2: Differentially expressed lncRNAs that correlate with patient survival, Table S3: Differentially expressed cancer-associated genes, Table S4: Dysregulated genes located within CNA regions associated with lncRNA dysregulation, Table S5: Interactions between microRNAs and lncRNAs, Table S6: The Cancer Genome Atlas (TCGA) patient identifiers.

## References

[1] Siegel R.L., Miller K.D., Jemal A. (2019). Cancer statistics, 2019. CA: Cancer J. Clin..

[2] Hussain M.H.A., MacVicar G.R., Etrylak D.P., Vaishampayan U., Lara P.N., Chatta G.S., Nanus D.M., Glode L.M., Trump D.L., Chen H. (2007). Trastuzumab, Paclitaxel, Carboplatin, and Gemcitabine in Advanced Human Epidermal Growth Factor Receptor-2/neu-Positive Urothelial Carcinoma: Results of a Multicenter Phase II National Cancer Institute Trial. J. Clin. Oncol..

[3] Ferre F., Colantoni A., Helmer-Citterich M. (2016). Revealing Protein-lncRNA Interaction. Brief. Bioinform..

[4] Sun M., Kraus W.L. (2015). From discovery to function: The expanding roles of long noncoding RNAs in Physiology and Disease. Endocr. Rev..

[5] Taheri M., Omrani M.D., Ghafouri-Fard S. (2018). Long Non-Coding RNA Expression in Bladder Cancer. Biophys. Rev..

[6] Zur Bruegge J., Einspanier R., Sharbati S. (2017). A Long Journey Ahead: Long Non-coding RNAs in Bacterial Infections. Front. Cell. Infect. Microbiol..

[7] Atala A. (2017). Re: Profiling of Long Non-Coding RNAs Identifies LINC00958 and LINC01296 as Candidate Oncogenes in Bladder Cancer. J. Urol..

[8] Cao X., Xu J., Yue D. (2018). LncRNA-SNHG16 Predicts Poor Prognosis and Promotes Tumor Proliferation through Epigenetically Silencing p21 in Bladder Cancer. Cancer Gene Ther..

[9] Liao X., Chen J., Liu Y., He A., Chen J., Zhang X., Lv Z., Wang F., Mei H. (2018). Knockdown of Long Noncoding RNA FGFR3- AS1 Induces Cell Proliferation Inhibition, Apoptosis and Motility Reduction in Bladder Cancer. Cancer Biomark..

[10] Wang H., Niu L., Jiang S., Zhai J., Wang P., Kong F., Jin X. (2016). Comprehensive Analysis of Aberrantly Expressed Profiles of lncRNAs and miRNAs with Associated ceRNA Network in Muscle-Invasive Bladder Cancer. Oncotarget.

[11] Seitz A.K., Christensen L.L., Christensen E., Faarkrog K., Ostenfeld M.S., Hedegaard J., Nordentoft I., Nielsen M.M., Palmfeldt J., Thomson M. (2017). Profiling of Long Non-Coding RNAs Identifies LINC00958 and LINC01296 as Candidate Oncogenes in Bladder Cancer. Sci. Rep..

[12] Paraskevopoulou M.D., Vlachos I.S., Karagkouni D., Georgakilas G., Kanellos I., Vergoulis T., Zagganas K., Tsanakas P., Floros E., Dalamagas T. (2016). DIANA-LncBase v2: Indexing microRNA Targets on Non-Coding Transcripts. Nucleic. Acids Res..

[13] Kim J.W., Botvinnik O.B., Abudayyeh O., Birger C., Rosenbluh J., Shrestha Y., Abazeed M.E., Hammerman P.S., DiCara D., Konieczkowski D.J. (2016). Characterizing Genomic Alterations in Cancer by Complementary Functional Associations. Nat. Biotechnol..

[14] Sondka Z., Bamford S., Cole C.G., Ward S.A., Dunham I., Forbes S.A. (2018). The COSMIC Cancer Gene Census: Describing genetic Dysfunction across all Human Cancers. Nat. Rev. Cancer.

[15] Anaya J. (2016). OncoLnc: Linking TCGA Survival Data to mRNAs, miRNAs, and lncRNAs. PeerJ. Computer Science.

[16] Paraskevopoulou M.D., Hatzigeorgiou A.G. (2016). Analyzing MiRNA-LncRNA Interactions. Methods Mol. Biol.

[17] Lin C., Yang L. (2018). Long Noncoding RNA in Cancer: Wiring Signaling Circuitry. Trends Cell Biol..

[18] Wen L., Zhang X., Bian J., Han L., Huang H., He M., Wei M., Wang P. (2019). The Long Non-Coding RNA LINC00460 Predicts the Prognosis and Promotes the Proliferation and Migration of Cells in Bladder Urothelial Carcinoma. Oncol. Lett..

[19] Volders P.-J., Anckaert J., Verheggen K., Nuytens K., Martens L., Mestdagh P., Vandesompele J. (2019). LNCipedia 5: Towards a Reference Set of Human Long Non-Coding RNAs. Nucleic. Acids Res..

[20] Avgeris M., Tsilimantou A., Levis P.K., Rampias T., Papadimitriou M.-A., Panoutsopoulou K., Stravodimos K., Scorilas A. (2019). Unraveling UCA1 lncRNA Prognostic Utility in Urothelial Bladder Cancer. Carcinogenesis.

[21] Jiang B., Su H., Yuan J., Zhao H., Xia W., Zha Z., Wu B., Liu Z. (2018). Identification of Oncogenic Long Noncoding RNA SNHG12 and DUXAP8 in Human Bladder Cancer through a Comprehensive Profiling Analysis. Biomed. Pharmacother..

[22] Richter J., Jiang F., Görög J.-P., Sartorius G., Egenter C., Gasser T.C., Moch H., Mihatsch M.j., Sauter g. (1997). Marked Genetic Differences between Stage pTa and Stage pT1 Papillary Bladder Cancer Detected by Comparative Genomic Hybridization. Cancer Res..

[23] Qin S.-L., Chen X.-J., Xu X., Shou J.-Z., Bi X.-G., Ji L., Han Y.-L., Cai Y., Wei F., Ma J.-H. (2006). Detection of Chromosomal Alterations in Bladder Transitional Cell Carcinomas from Northern China by Comparative Genomic Hybridization. Cancer Lett..

[24] Chang W.Y.-H., Cairns P., Schoenberg M.P., Polascik T.J., Sidransky D. (1995). Novel Suppressor Loci on Chromosome 14q in Primary Bladder Cancer. Cancer Res..

[25] Robinson M.D., McCarthy D.J., Smyth G.K. (2010). edgeR: A Bioconductor package for Differential Expression Analysis of Digital Gene Expression Data. Bioinformatics.

[26] Subramanian A., Tamayo P., Mootha V.K., Mukherjee S., Ebert B.L., Gillette M.A., Paulovich A., Pomeroy S.L., Golub T.R., Lander E.S. (2005). Gene Set Enrichment Analysis: A Knowledge-Based Approach for Interpreting Genome-Wide Expression Profiles. Proc. Natl. Acad. Sci. USA.

[27] Ni Y., Song C., Jin S., Chen Z., Ni M., Han L., Wu J., Jin Y. (2018). Gene Set Enrichment Analysis: A Genome-Wide Expression Profile-Based Strategy for Discovering Functional microRNA-Disease Relationships. J. Int. Med. Res..

[28] Li W.T., Zheng H., Nguyen V., Wang-Rodriguez J., Ongkeko W.M. (2018). Functional Genomics Profiling of Bladder Urothelial Carcinoma MicroRNAome as a Potential Biomarker. Neoplasia.

