# Supplementary material for: The Landscape of Long Non-Coding RNA Dysregulation and Clinical Relevance in Muscle Invasive Bladder Urothelial Carcinoma
Source: Cancers (Basel). 2019 Dec 2;11(12):1919. doi: 10.3390/cancers11121919 (PMC6966549; doi:10.3390/cancers11121919)
Supplement: Supplementary file 1 [file cancers-11-01919-s001.zip › cancers-640001-suppl.-final/Supplementary Figures/Supplementary Figures Legends.docx]

Article

The Landscape of Long Non-Coding RNA Dysregulation and Clinical Relevance in Muscle Invasive Bladder Urothelial Carcinoma

Haotian Shen ^1,†^, Lindsay M. Wong ^1,†^, Wei Tse Li ^1,†^, Megan Chu ^1^, Rachel A. High ^2,3^, Eric Y. Chang ^2,3^, Jessica Wang-Rodriguez ^4,5^ and Weg M. Ongkeko ^1,^*

**Figure S1.** Comparison of expression levels between lncRNA and mRNA transcripts. (**A**) Magnified and (**B**) normal view of boxplots that compare lncRNA expression to expression of genes implicated in BLCA.

**Figure S2.** Survival correlation with lncRNA expression. Kaplan Meier plots of (**A**) significantly downregulated and (**B**) significantly upregulated lncRNAs that correlated with patient survival and clinical variables.

**Figure S3.** Canonical pathways filtered for cancer-associated pathways and immune-associated pathways (C2) from gene set enrichment analysis (Nominal *p* < 0.05). Barplots showing (**A**) significantly downregulated and (**B**) significantly upregulated lncRNAs correlated with fewer than 15 cancer-associated and immune-associated pathways.

**Figure S4.** Significant oncogenic signatures (C6) from GSEA (Nominal *p* < 0.05). Barplots showing (**A**) significantly downregulated lncRNAs and (**B**) significantly upregulated lncRNAs correlated with fewer than 10 oncogenic signatures. All bars extending towards the left indicate negative enrichment of pathway activity to lncRNA expression, such that higher pathway activity corresponds to lower lncRNA expression. The reverse is true for bars extending towards the right.
