# Supplementary figures and images for: The Landscape of Long Non-Coding RNA Dysregulation and Clinical Relevance in Muscle Invasive Bladder Urothelial Carcinoma
Source: Cancers (Basel). 2019 Dec 2;11(12):1919. doi: 10.3390/cancers11121919 (PMC6966549; doi:10.3390/cancers11121919)

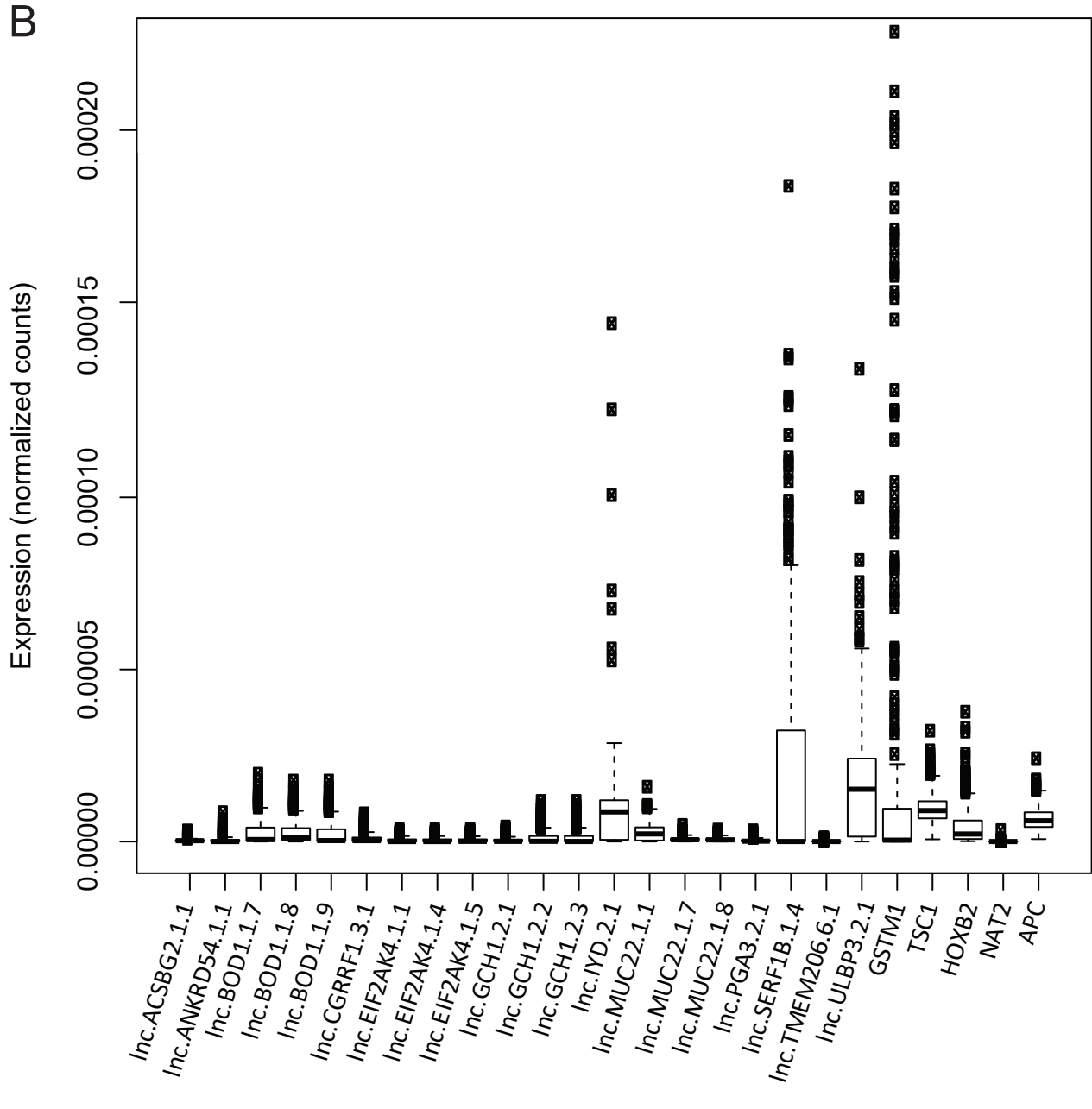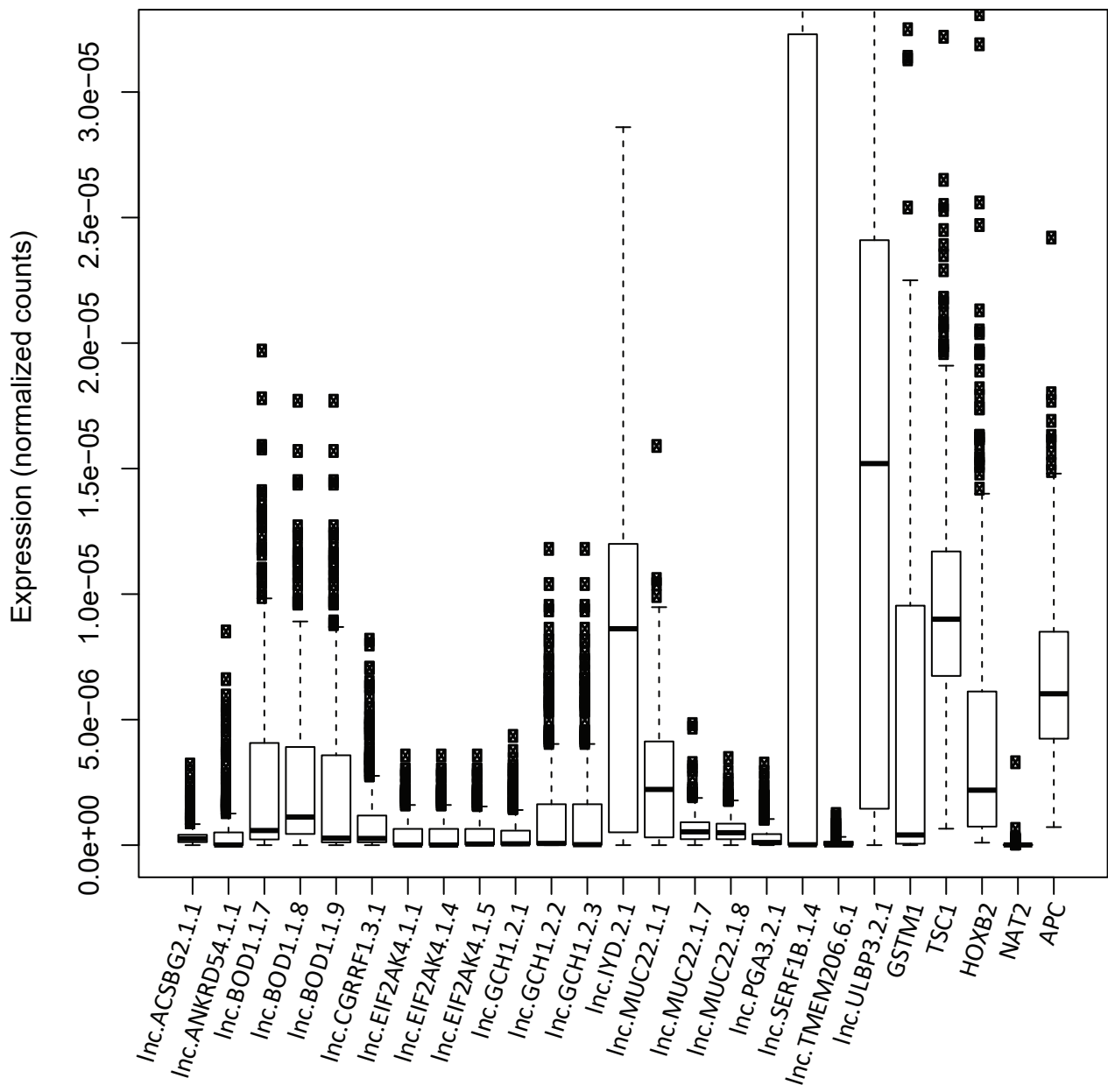

Supplement: Supplementary file 1 [file cancers-11-01919-s001.zip › cancers-640001-suppl.-final/Supplementary Figures/Figure S1. lncRNA Expression vs. Gene Expression Comparison.pdf]

A

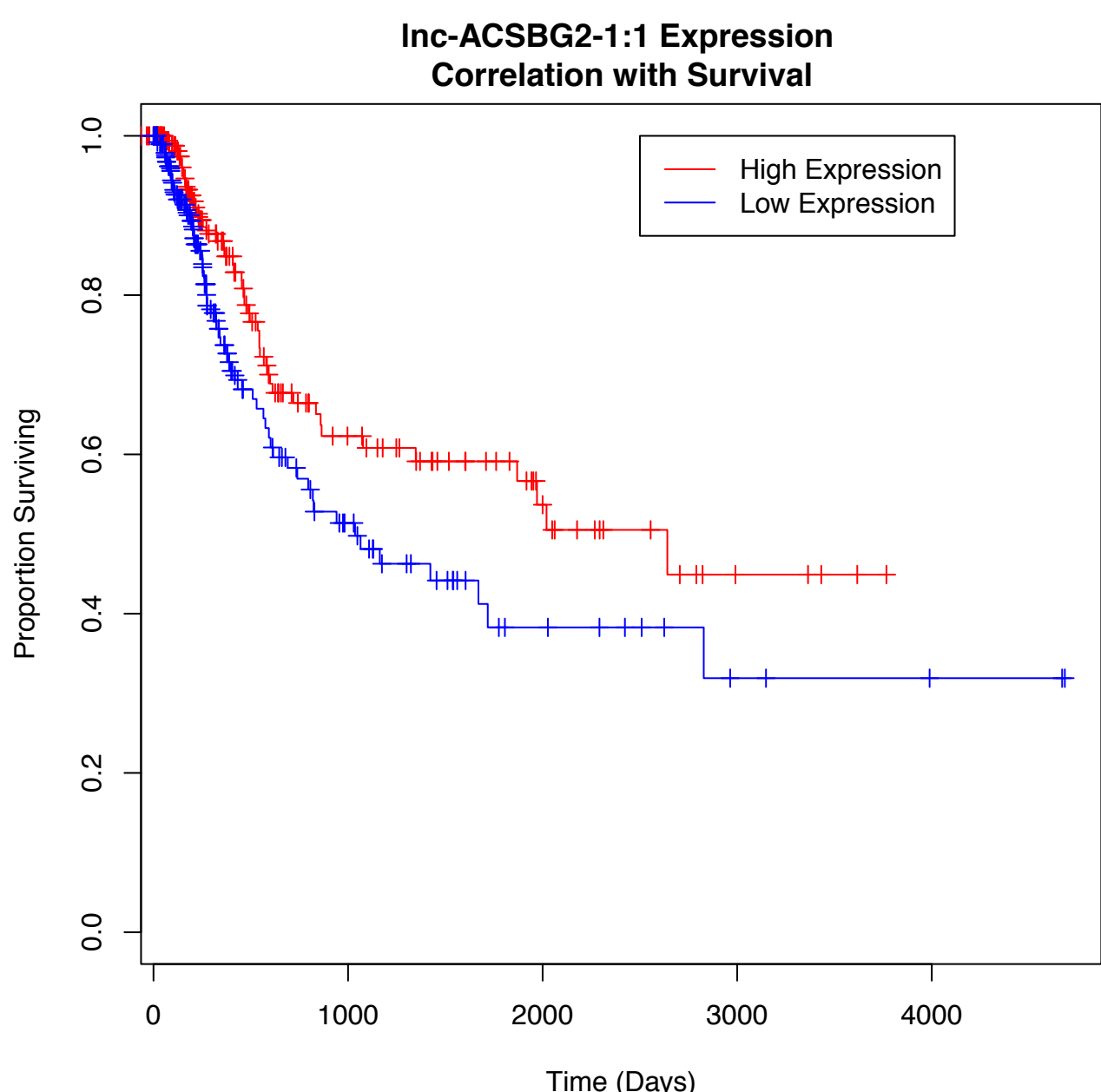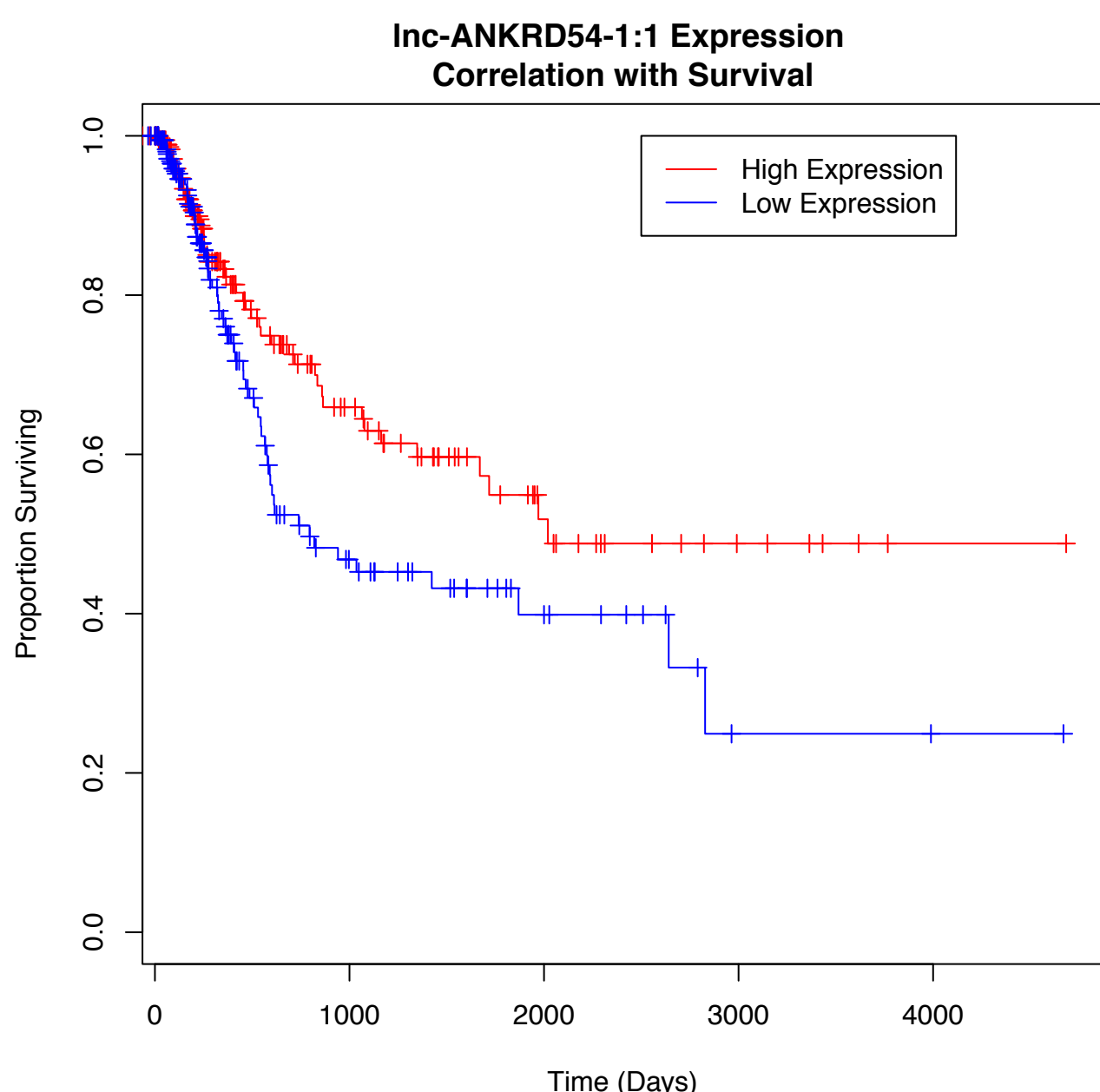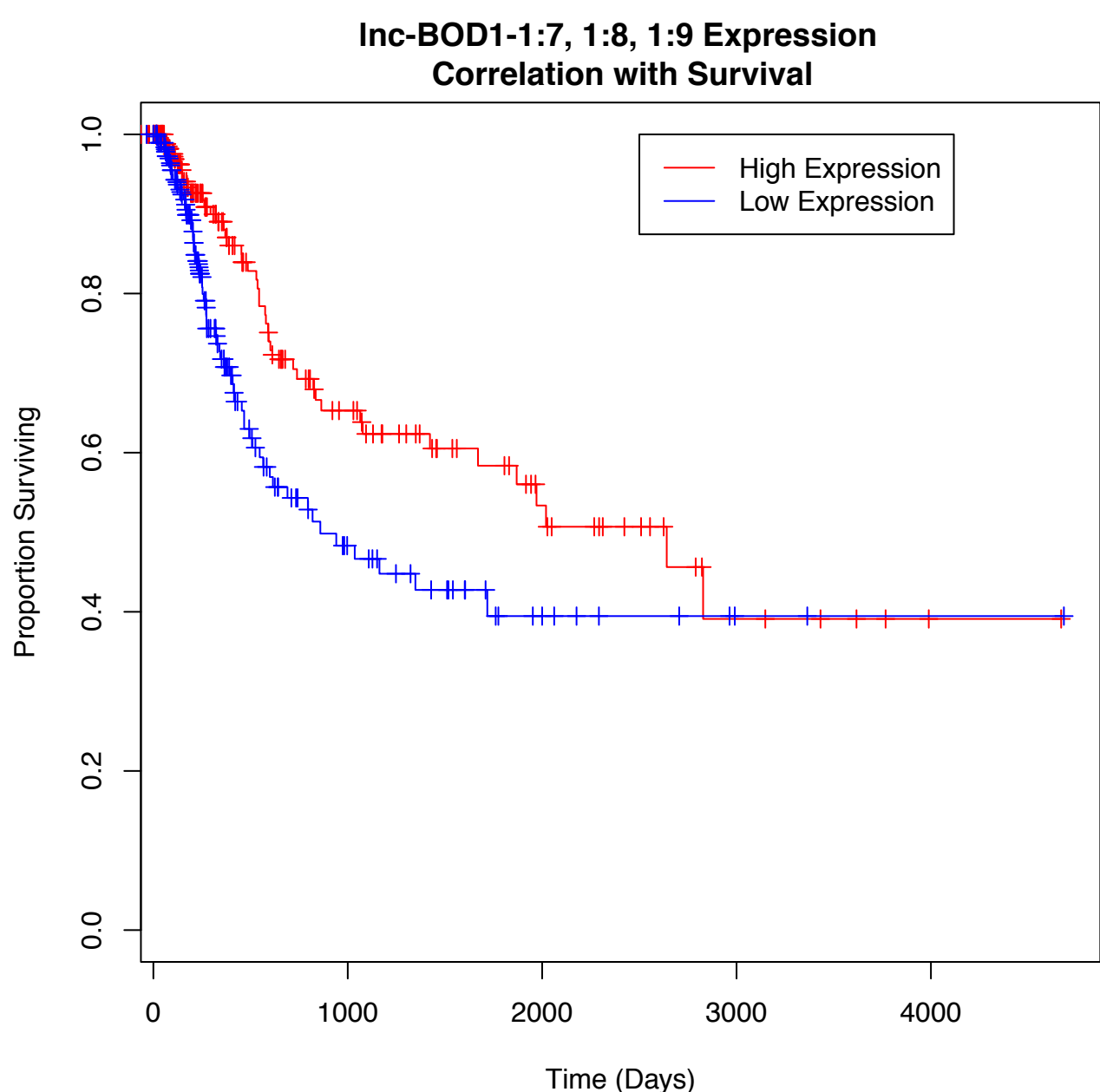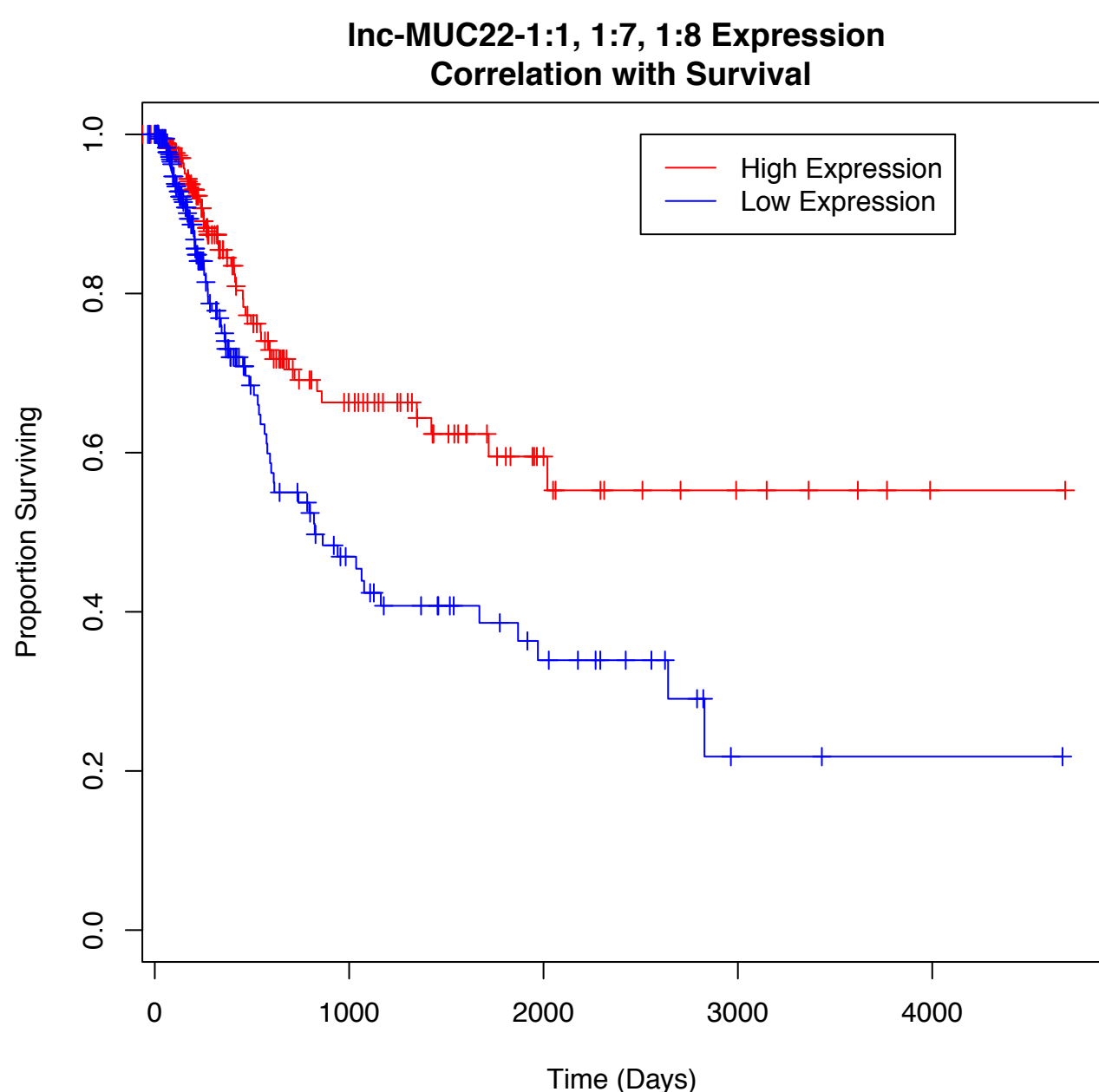

B

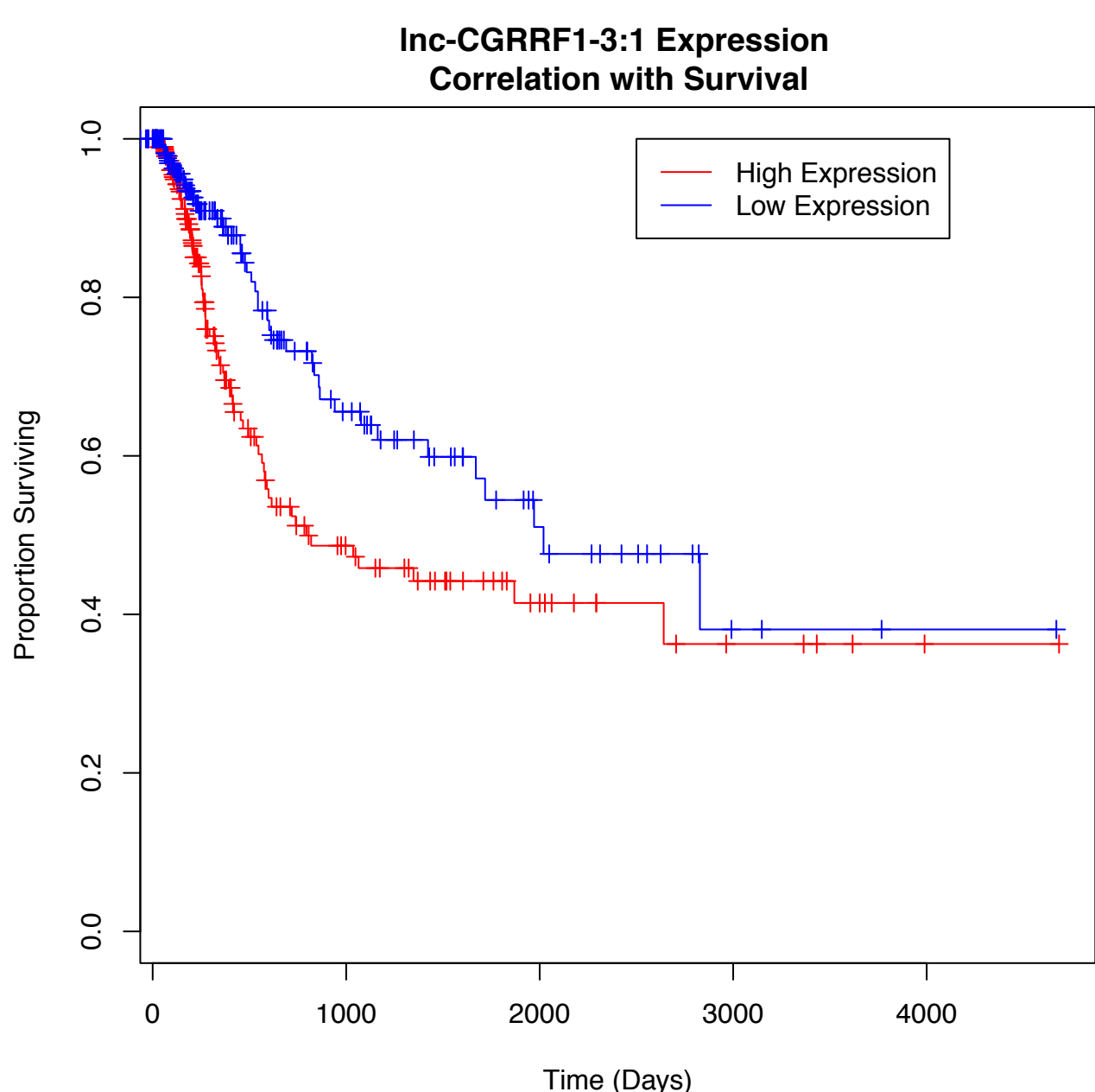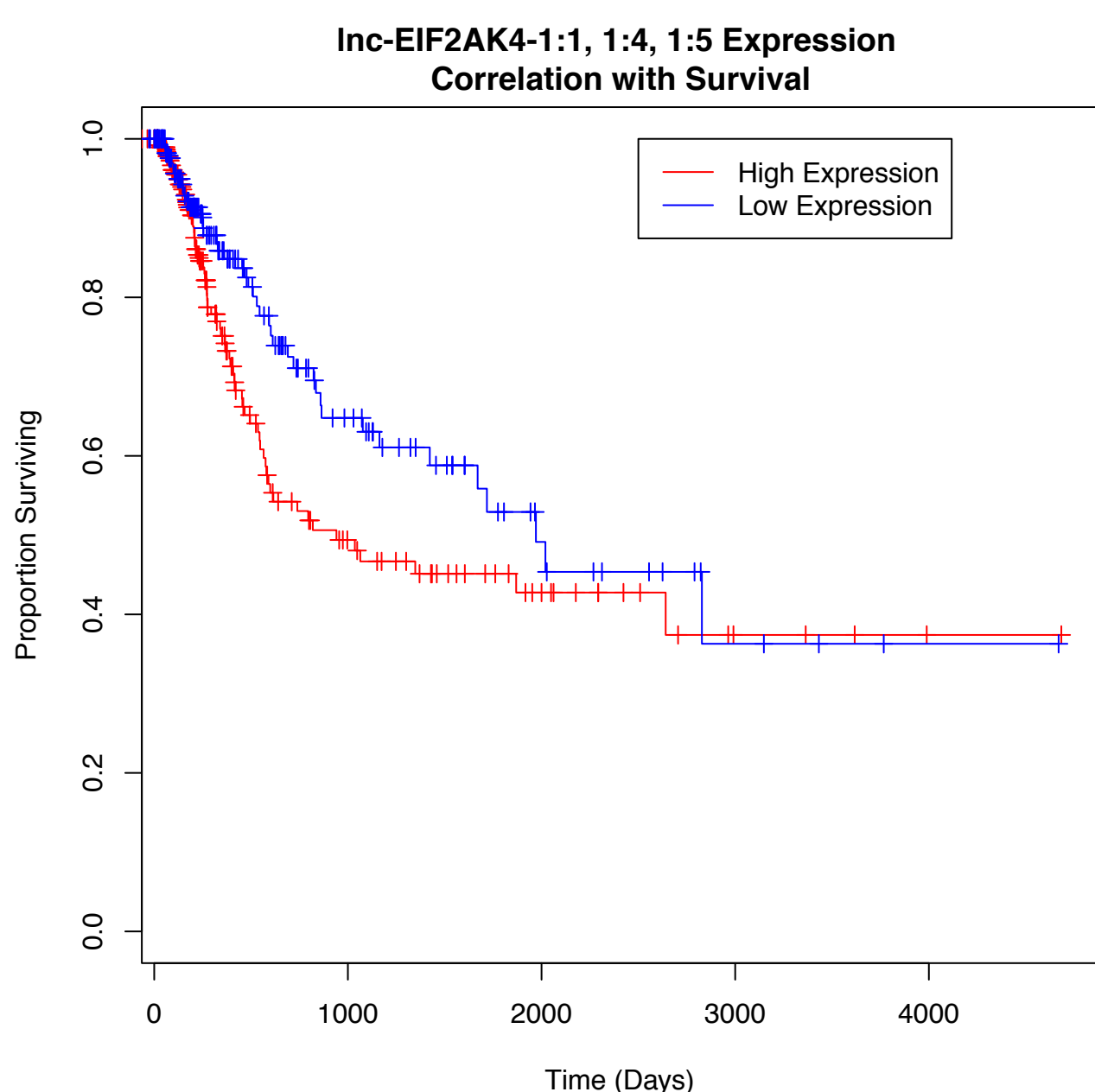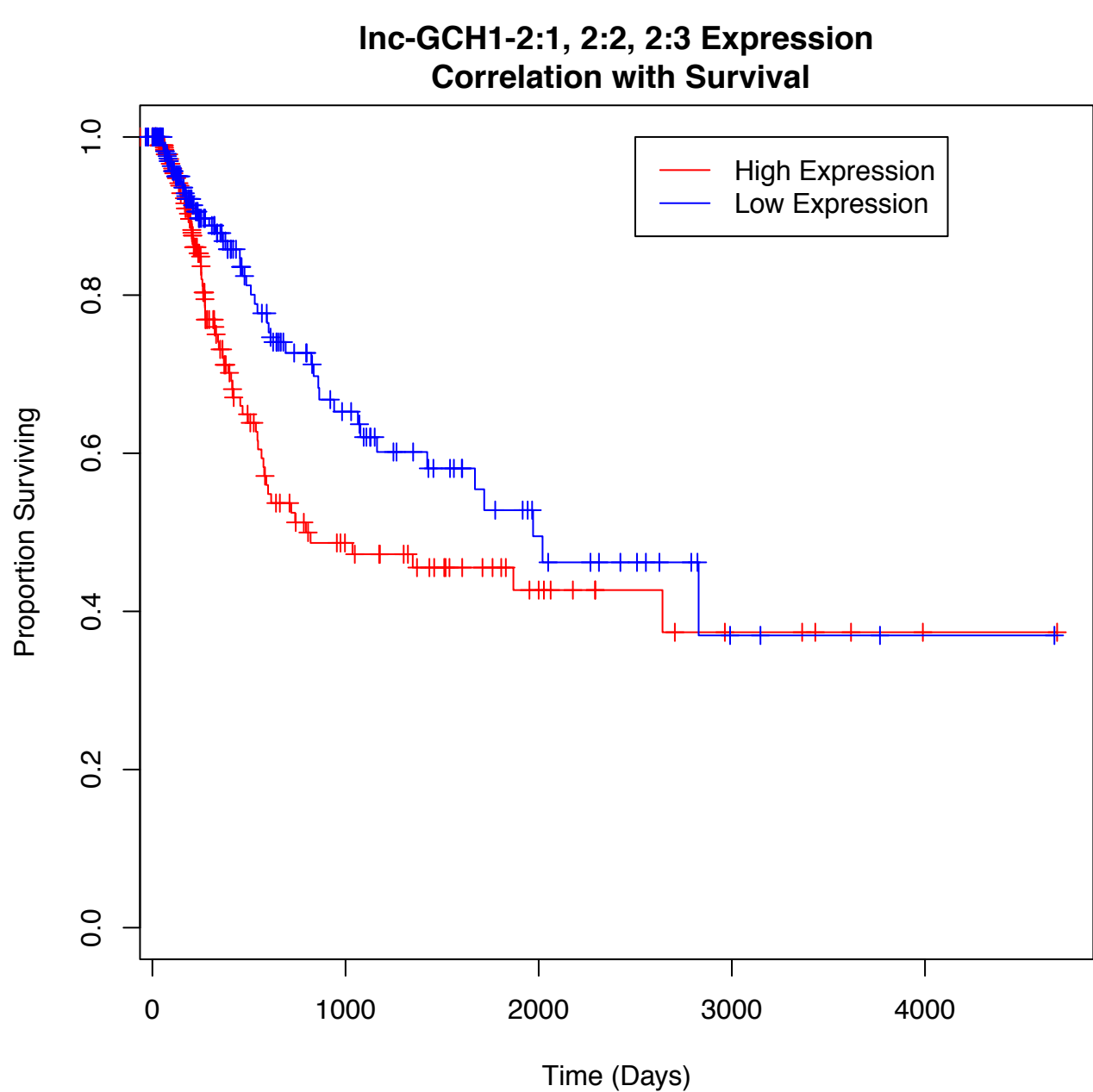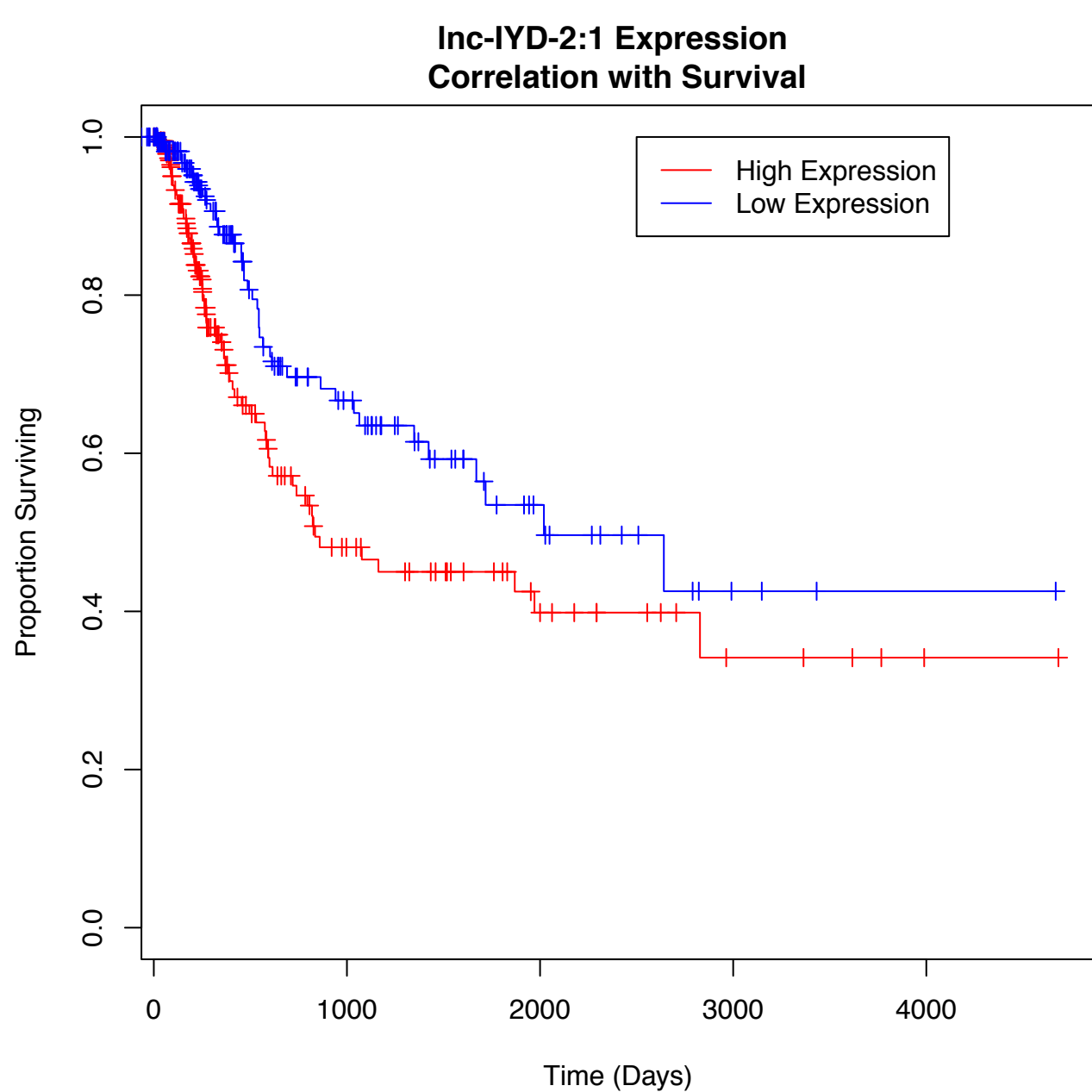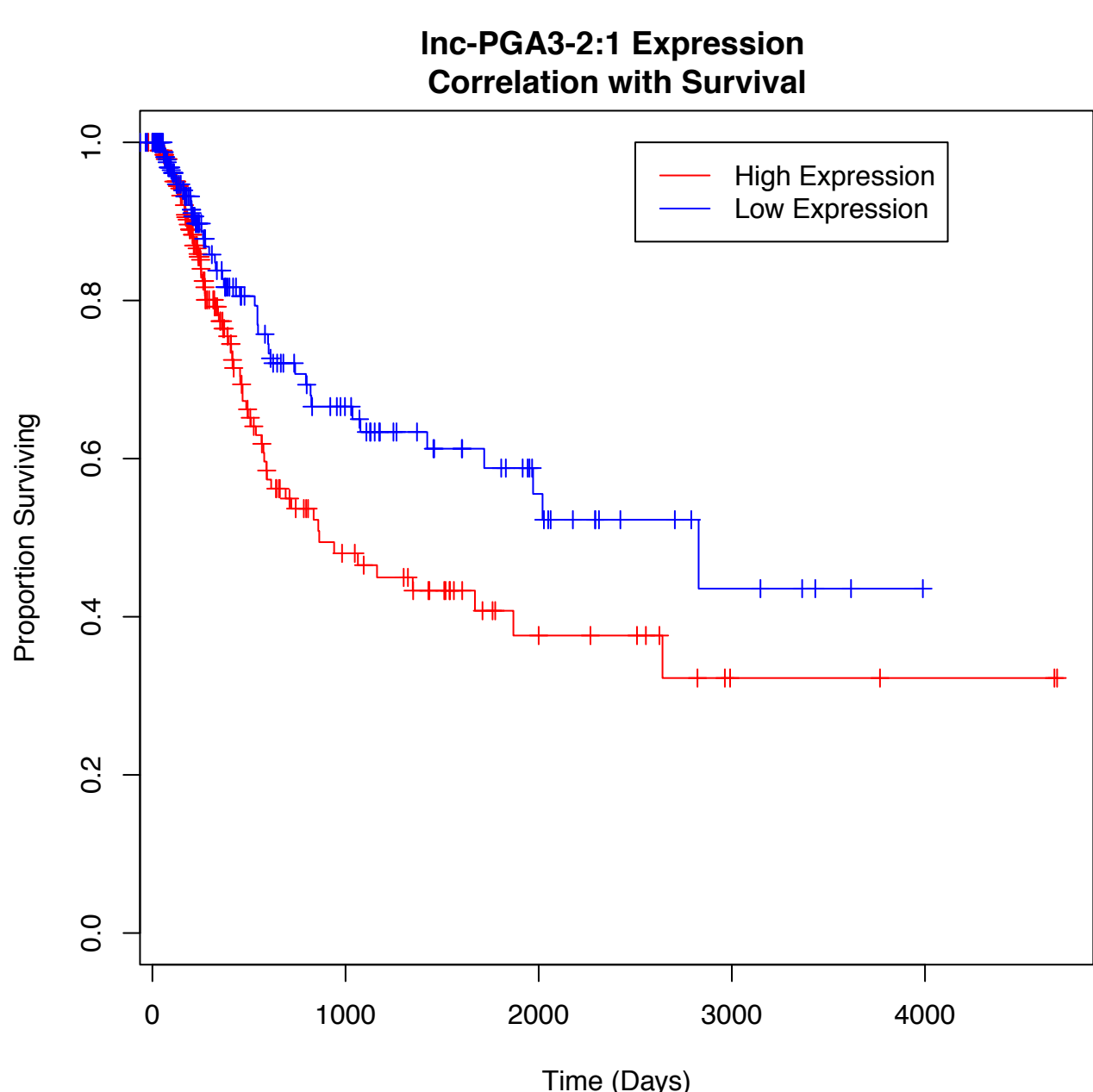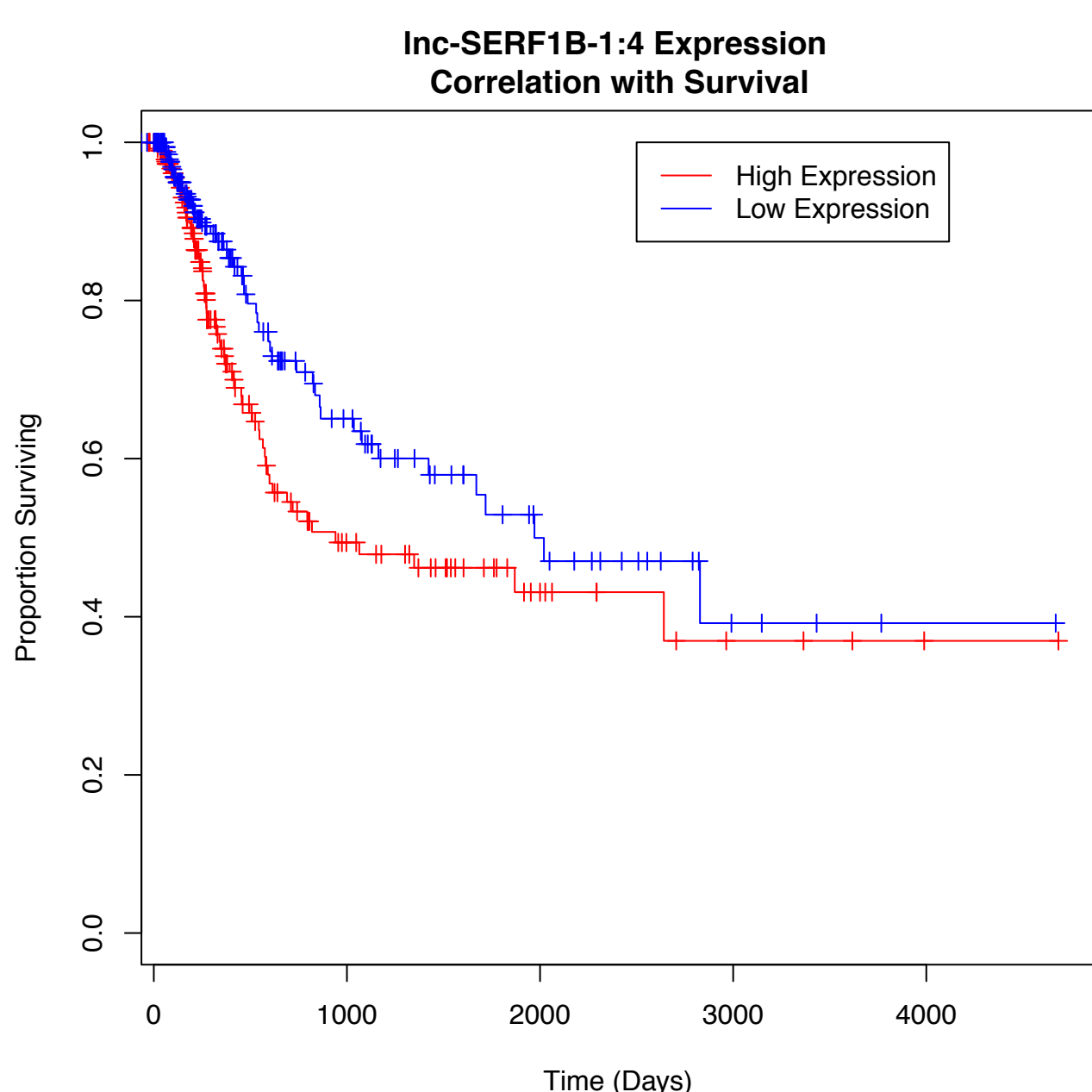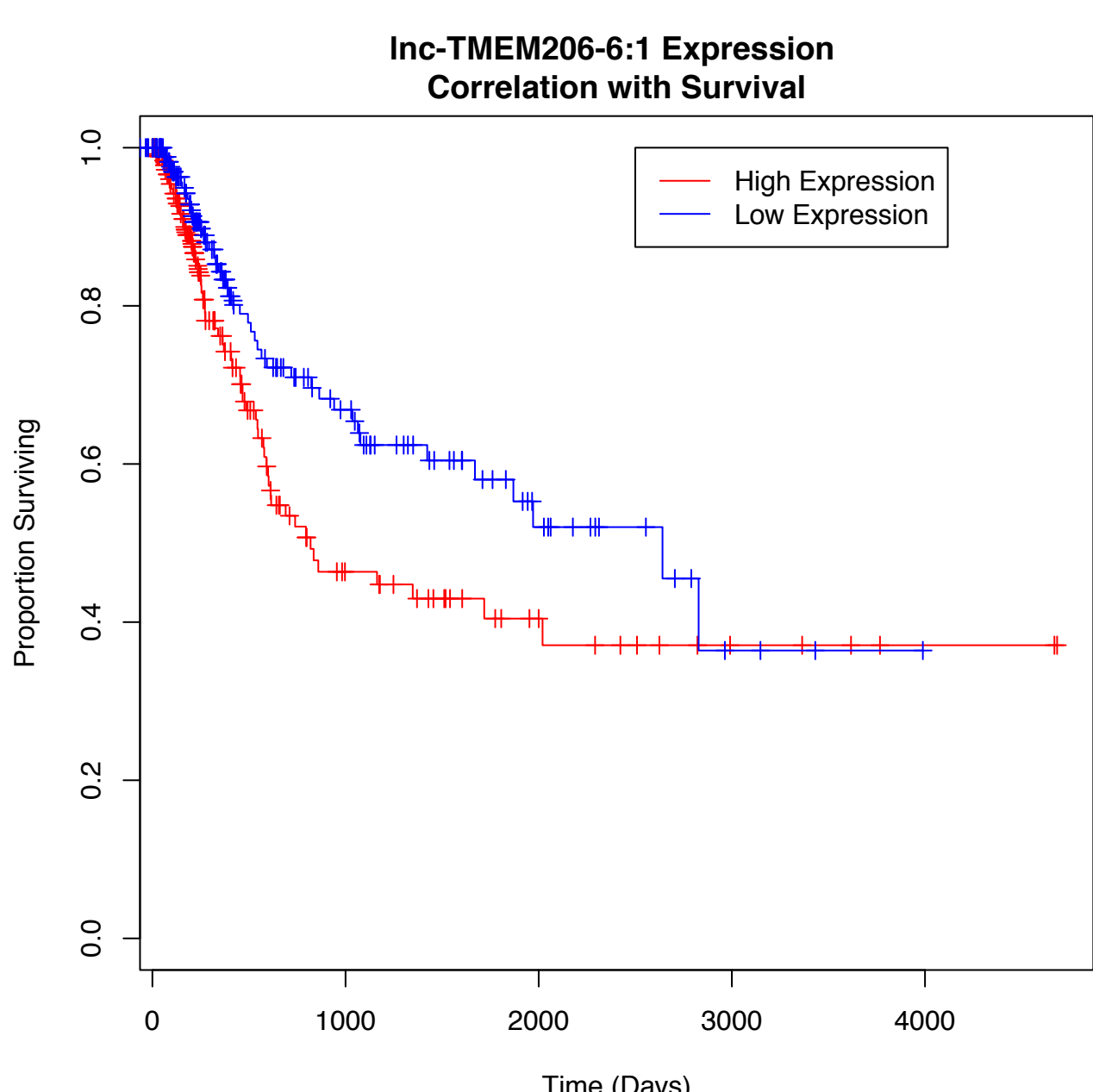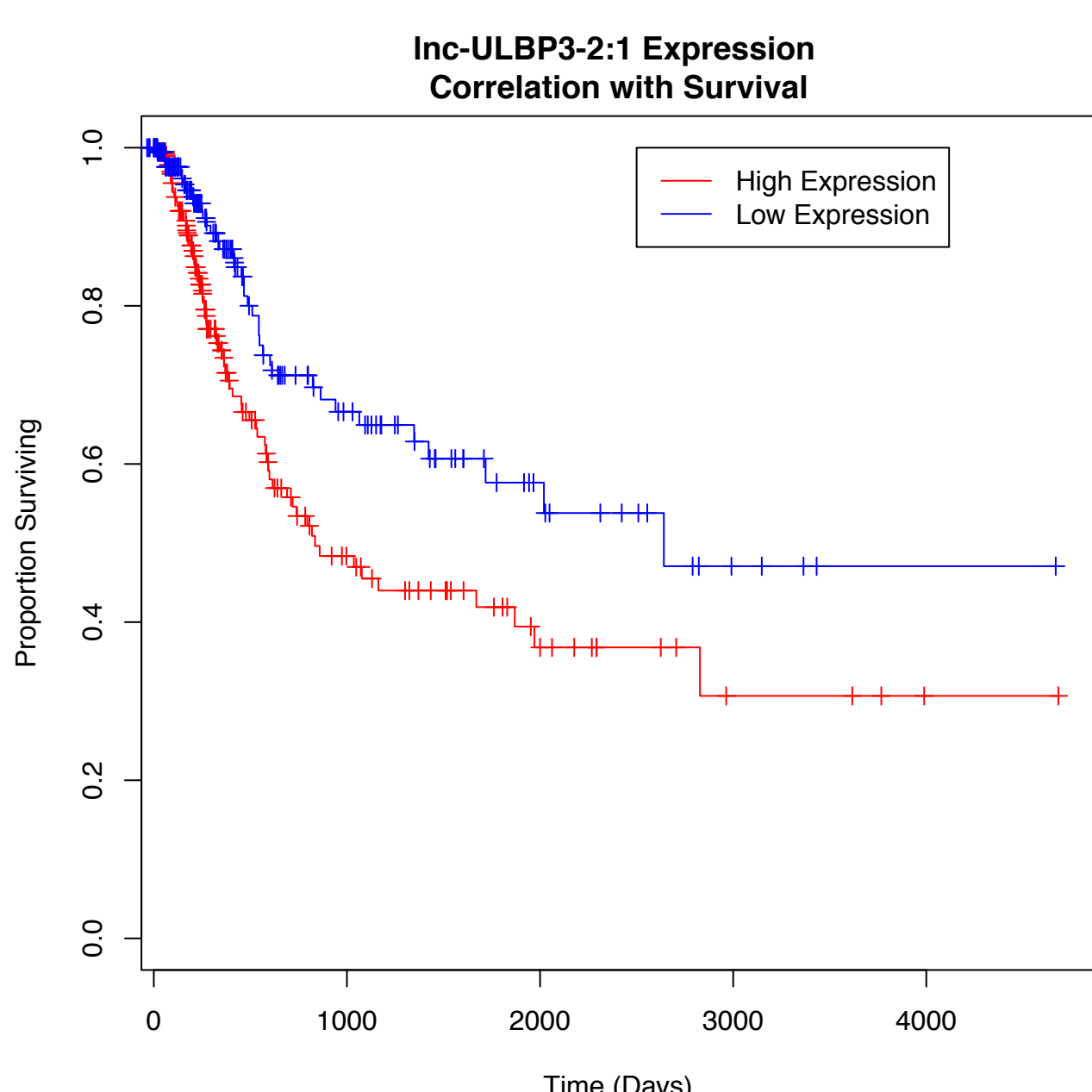

Supplement: Supplementary file 1 [file cancers-11-01919-s001.zip › cancers-640001-suppl.-final/Supplementary Figures/Figure S2. Kaplan Meier Graphs (Survival).pdf]

A

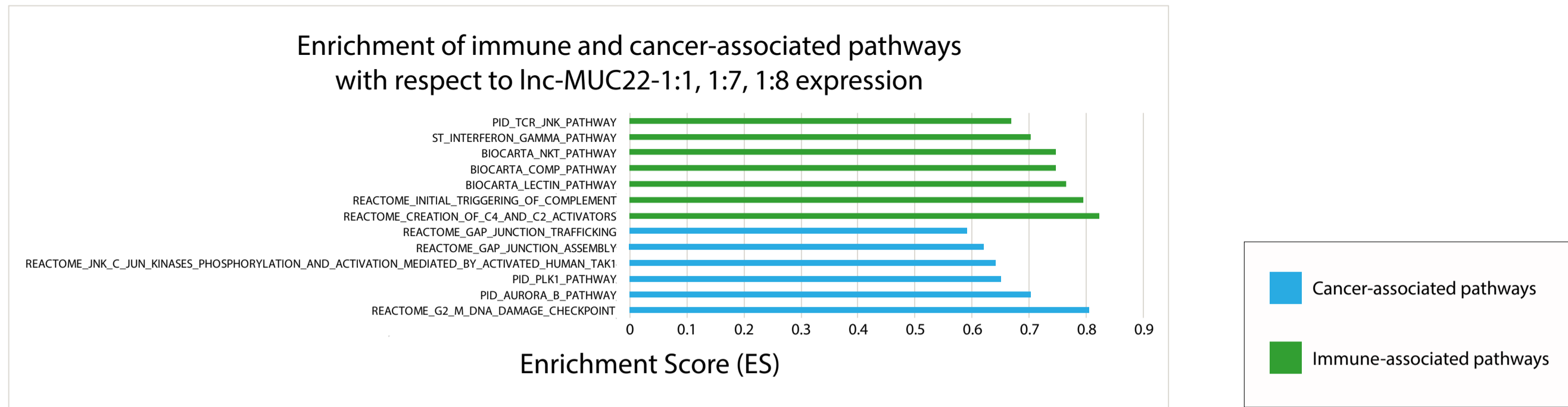

B

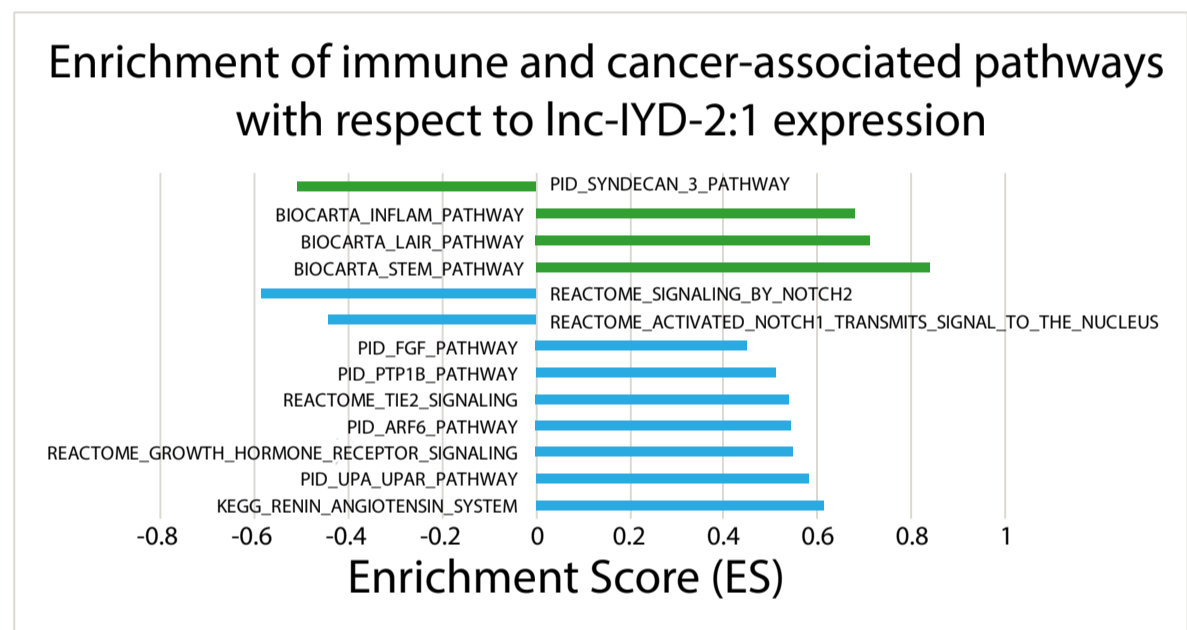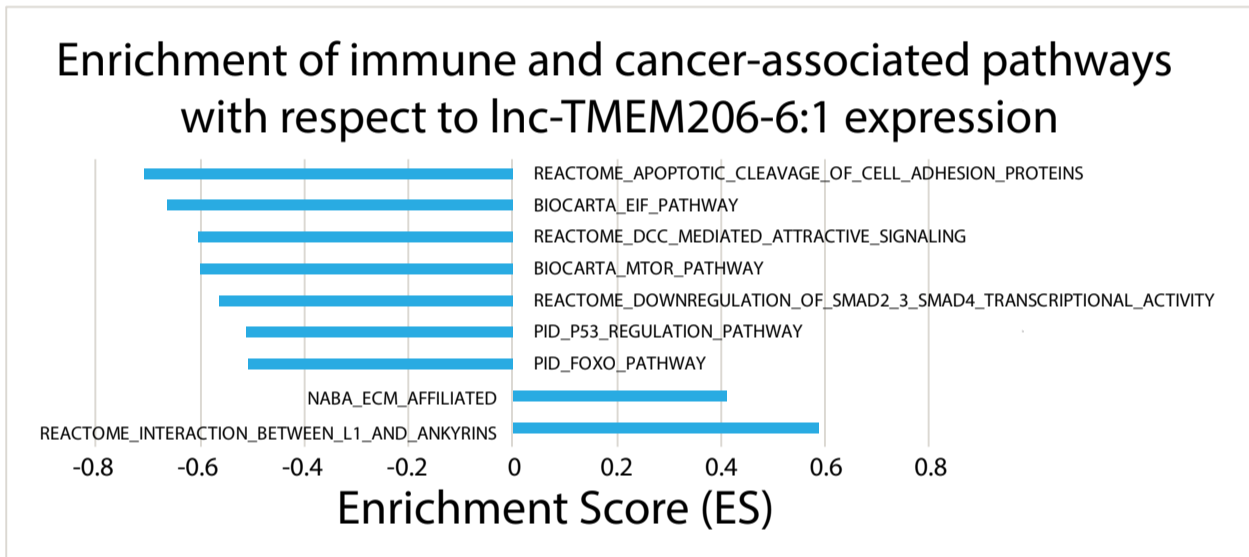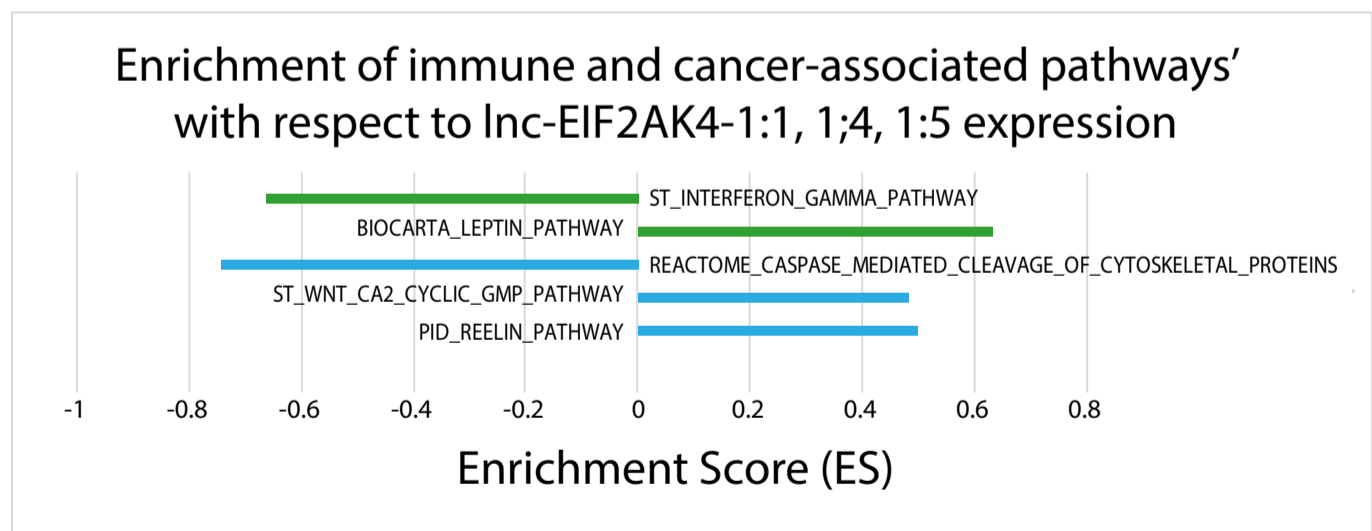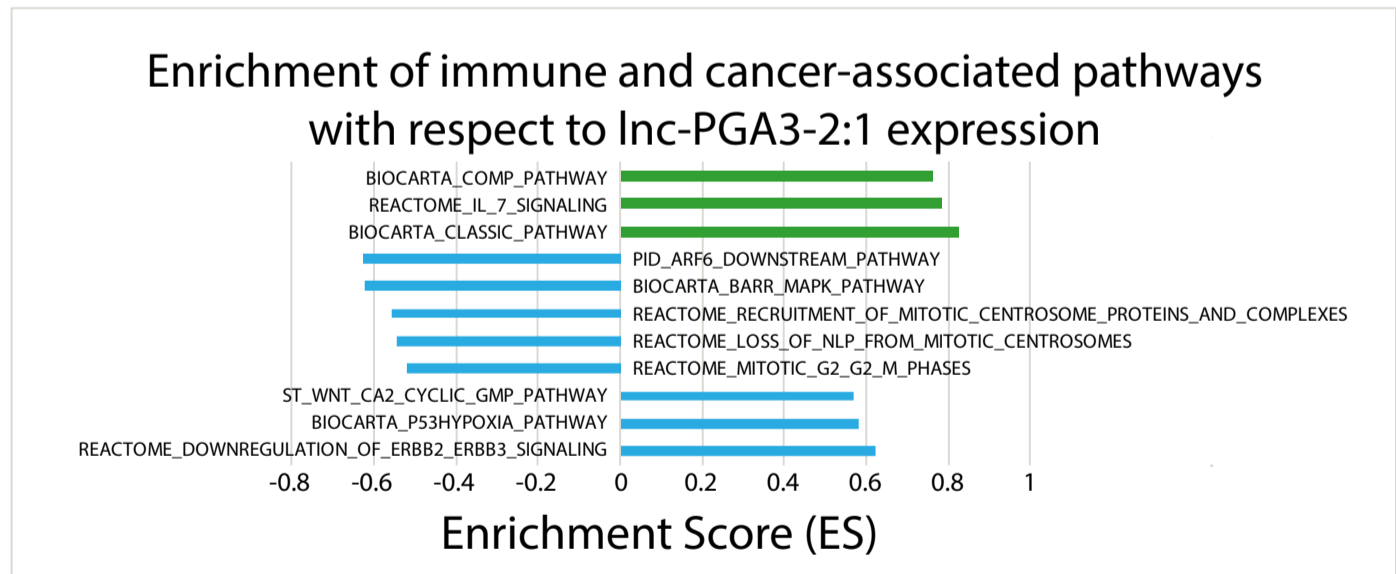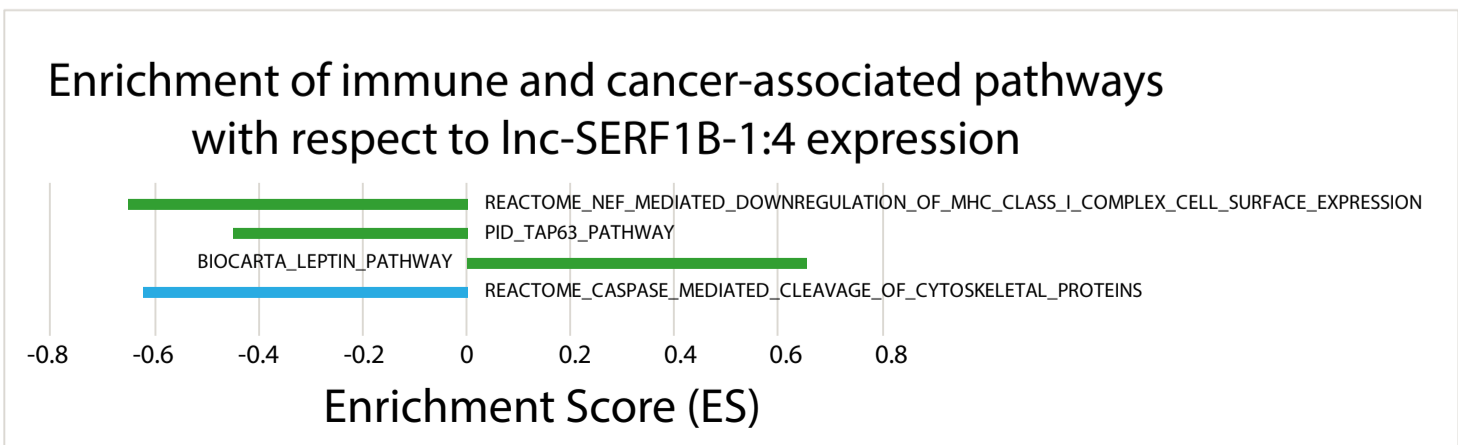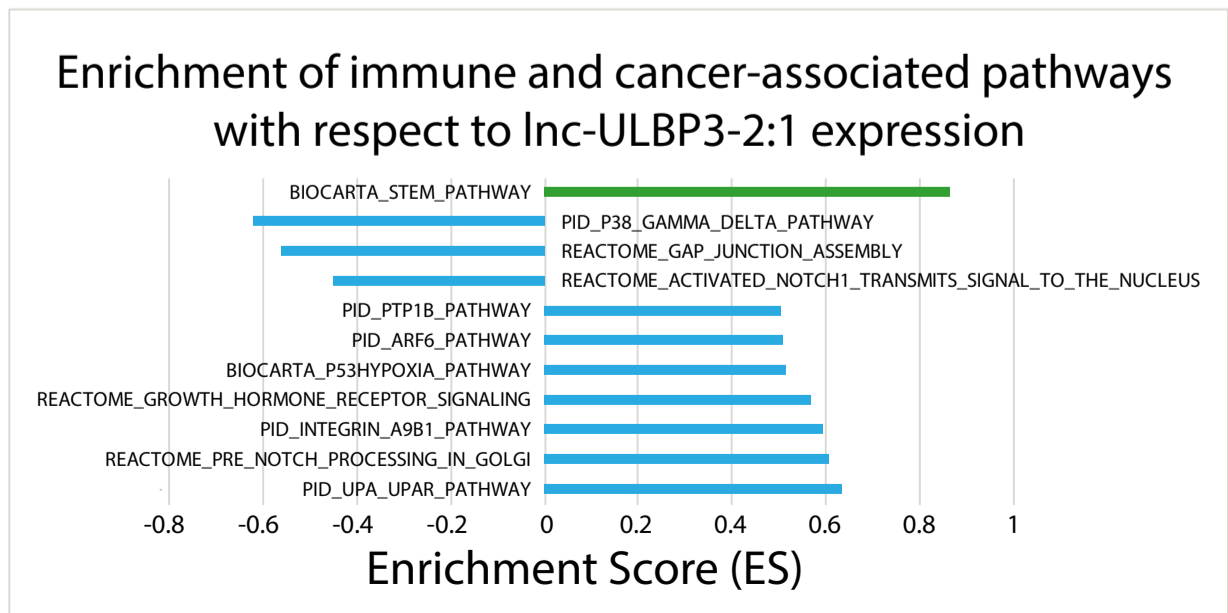

Supplement: Supplementary file 1 [file cancers-11-01919-s001.zip › cancers-640001-suppl.-final/Supplementary Figures/Figure S3. C2 Bar Graphs.pdf]

A

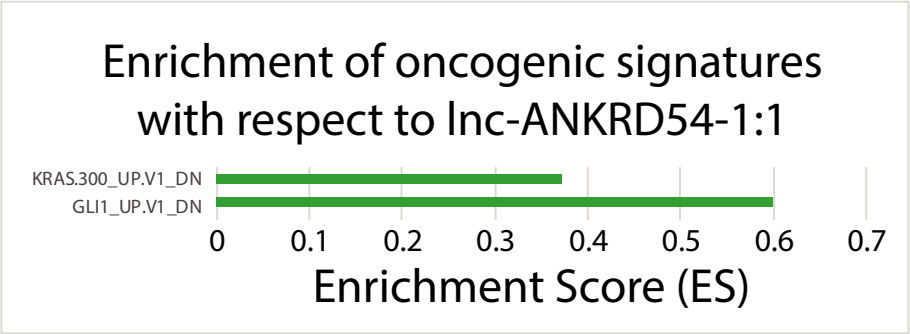

B

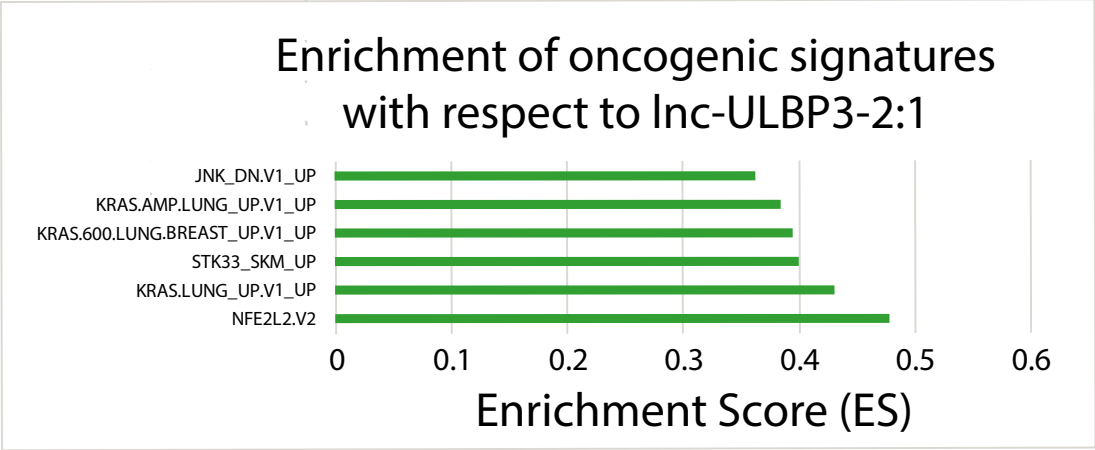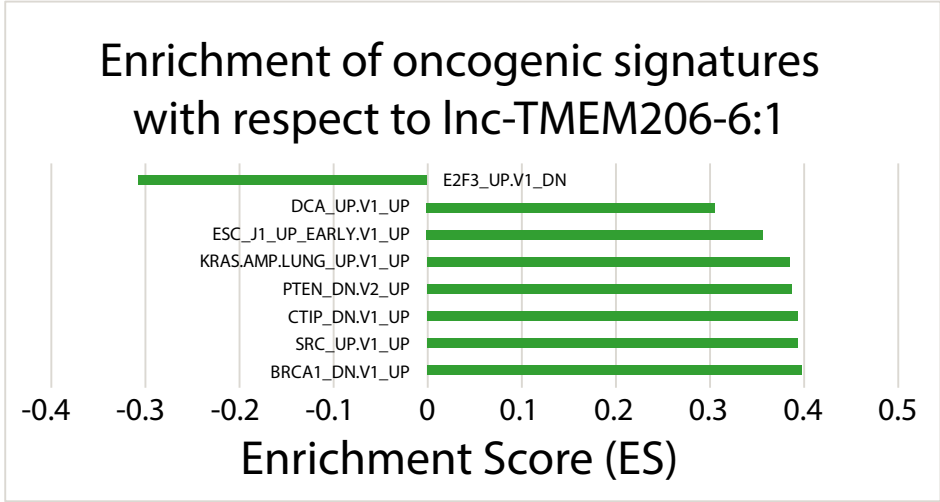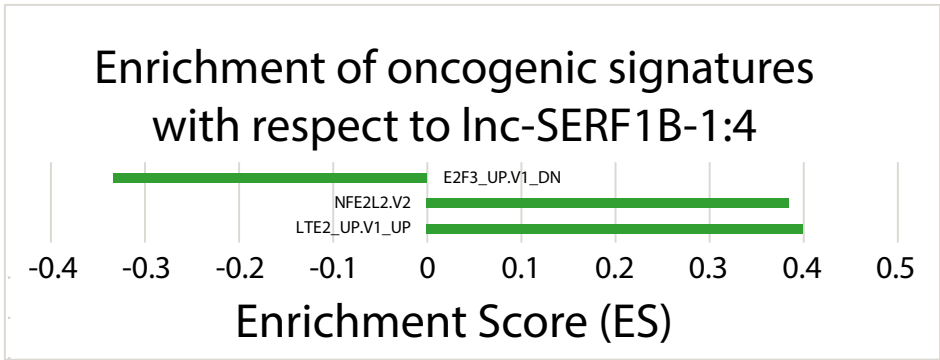

Supplement: Supplementary file 1 [file cancers-11-01919-s001.zip › cancers-640001-suppl.-final/Supplementary Figures/Figure S4. C6 Bar Graphs.pdf]
